# Body mass index and risk of obesity‐related conditions in a cohort of 2.9 million people: Evidence from a UK primary care database

**DOI:** 10.1002/osp4.474

**Published:** 2020-12-24

**Authors:** Christiane L. Haase, Kirsten T. Eriksen, Sandra Lopes, Altynai Satylganova, Volker Schnecke, Phil McEwan

**Affiliations:** ^1^ Novo Nordisk A/S Søborg Denmark; ^2^ Health Economics and Outcomes Research Ltd Cardiff UK

**Keywords:** body mass index (BMI), obesity, outcomes, risk factors

## Abstract

**Objective:**

Obesity rates in the United Kingdom are some of the highest in Western Europe, with considerable clinical and societal impacts. Obesity is associated with type 2 diabetes (T2D), osteoarthritis, cardiovascular disease, and increased mortality; however, relatively few studies have examined the occurrence of multiple obesity‐related outcomes in the same patient population. This study was designed to examine the associations between body mass index (BMI) and a broad range of obesity‐related conditions in the same large cohort from a UK‐representative primary care database.

**Methods:**

Demographic data and diagnosis codes were extracted from the Clinical Practice Research Datalink GOLD database in January 2019. Adults registered for ≥ 3 years were grouped by BMI, with BMI 18.5–24.9 kg/m^2^ as reference group. Associations between BMI and 12 obesity‐related outcomes were estimated using Cox proportional hazard models, adjusted for age, sex, and smoking.

**Results:**

More than 2.9 million individuals were included in the analyses and were followed up for occurrence of relevant outcomes for a median of 11.4 years during the study period. Generally, there was a stepwise increase in risk of all outcomes with higher BMI. Individuals with BMI 40.0–45.0 kg/m^2^ were at particularly high risk of sleep apnea (hazard ratio [95% confidence interval] vs. reference group: 19.8 [18.9–20.8]), T2D (12.4 [12.1–12.7]), heart failure (3.46 [3.35–3.57]), and hypertension (3.21 [3.15–3.26]).

**Conclusions:**

This study substantiates evidence linking higher BMI to higher risk of a range of serious health conditions, in a large, representative UK cohort. By focusing on obesity‐related conditions, this demonstrates the wider clinical impact and the healthcare burden of obesity, and highlights the vital importance of management, treatment approaches, and public health programs to mitigate the impact of this disease.

## 1 INTRODUCTION

In 2016, more than 1.9 billion adults worldwide were above healthy weight (body mass index [BMI] ≥ 25 kg/m^2^) and more than 650 million of these individuals were living with obesity (BMI ≥ 30 kg/m^2^).^1^ In line with this global trend, the prevalence of obesity has risen steadily in the United Kingdom, with a particularly sharp increase between 1993 and 2000.^2^ A report by the Organisation for Economic Co‐operation and Development using data from 2018 found that the UK overweight and obesity rates were some of the highest in Western Europe at 63% of the adult population^3^; according to 2017 data, 26% of adults in England were living with obesity.^4^

Across various observational studies, individuals with higher BMI were shown to be at higher risk of a range of chronic conditions, including sleep apnea,^5^ type 2 diabetes (T2D), gallbladder disease, and osteoarthritis,^6^ compared with those of healthy weight. In addition, higher BMI has been linked with higher incidence of cardiovascular conditions such as hypertension, dyslipidemia, stroke, myocardial infarction (MI), and coronary heart disease.^6^, ^7^ Cardiovascular disease accounts for a considerable proportion of obesity‐related mortality: a meta‐analysis estimated that, in 2015, approximately 4 million deaths worldwide were attributable to high BMI, of which 2.7 million were linked to cardiovascular disease and 0.9 million were linked to diabetes.^8^ Across studies, mortality has been shown to increase non‐linearly with increasing BMI.^9^, ^10^

The total cost to the UK National Health Service (NHS) of treating overweight, obesity and associated conditions was estimated at £6.1 billion in 2014–2015, and costs are projected to rise to £9.7 billion by 2050.^11^ Another projection of future obesity‐related healthcare costs in the United Kingdom suggested that treatment of comorbid conditions including T2D, heart disease, and stroke is likely to constitute a considerable proportion of this economic impact in the coming decades, with an estimated £2 billion annual excess spending on obesity‐related conditions by 2030.^12^ Obesity and its comorbidities impose not only direct treatment costs on healthcare systems,^13^ but also indirect costs on society, such as loss of work productivity.^14^

It is vital to assess further the association over time between obesity and other health conditions, to understand both the clinical impact on individuals living with overweight or obesity and the wider burden on healthcare systems. This study was designed to examine how a broad range of obesity‐related conditions and events associate with overweight or obesity, compared with healthy weight, in a large cohort considered generalizable to populations in everyday clinical practice. To allow longitudinal assessment of a large patient sample and inclusion of multiple relevant outcomes in the analyses, data from the well‐recognized Clinical Practice Research Datalink (CPRD) GOLD were used. CPRD GOLD is a large, population‐representative UK primary care database that links to secondary care data sets and is widely used in epidemiology research across a broad range of disease areas,^15^ including obesity.^16^

## 2 MATERIALS AND METHODS

### 2.1 Data sources and study population

This retrospective, longitudinal, observational cohort study used data from the CPRD GOLD,^17^ an ongoing database of anonymized primary care records from general practitioners in the United Kingdom that includes information on patient demographics, disease symptoms, laboratory test results, diagnoses, treatment, health‐related behaviors and referrals to secondary care. The individuals in CPRD GOLD are representative of the overall UK adult population in terms of age, sex and ethnicity.^18^

**FIGURE 1:**
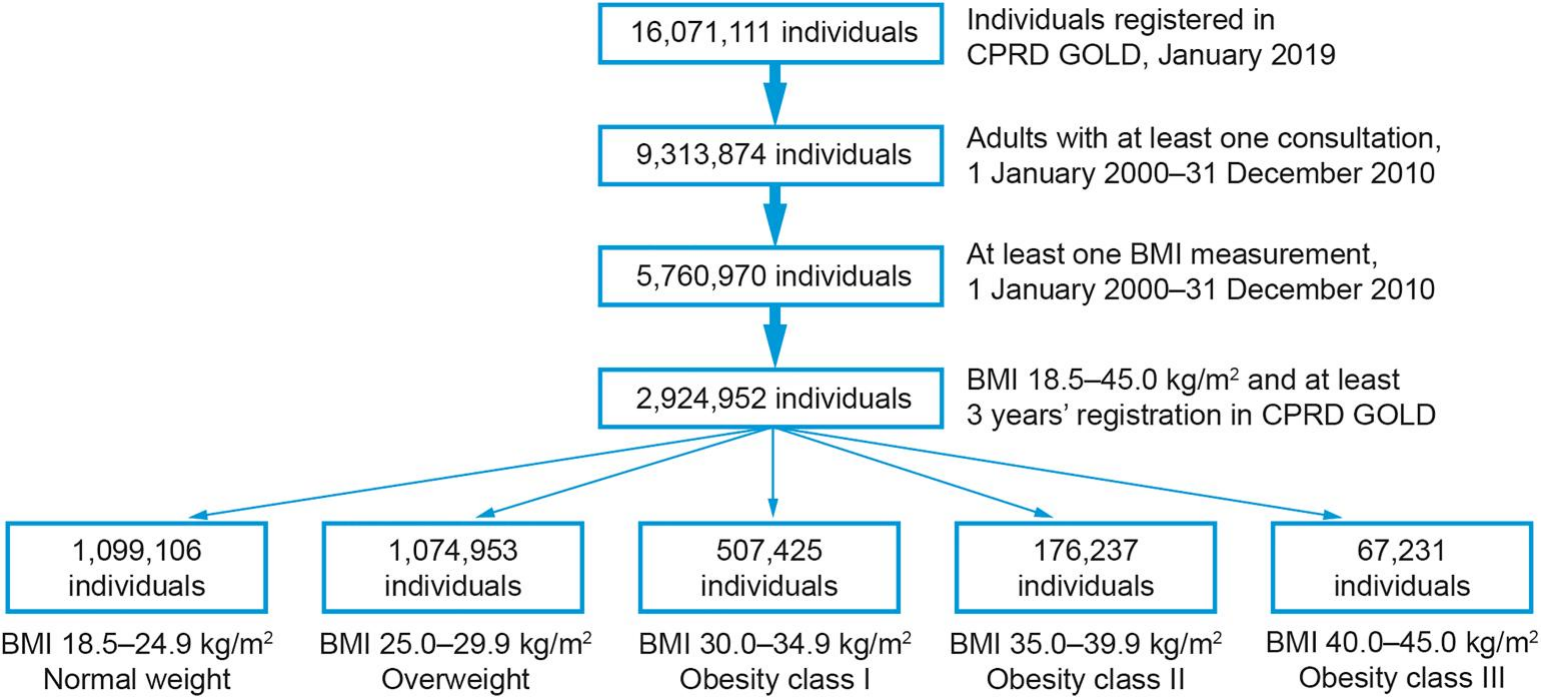
Study flow diagram. BMI, body mass index; CPRD, Clinical Practice Research Datalink

**FIGURE 2:**
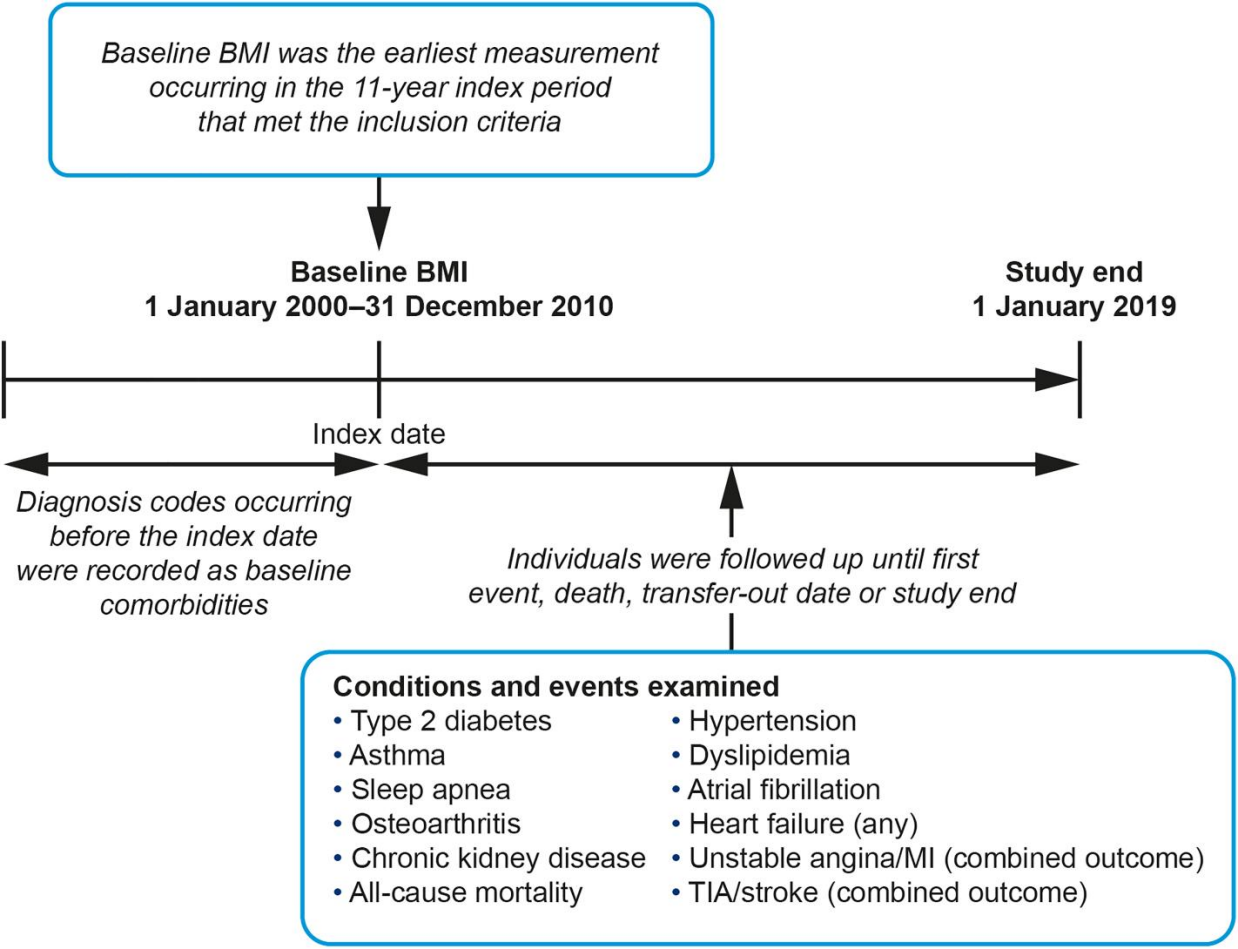
Study design. BMI, body mass index; MI, myocardial infarction; TIA, transient ischemic attack

Data were extracted from the CPRD GOLD database in January 2019 and merged with data from Hospital Episode Statistics and death registration data from the Office for National Statistics. This allowed information originating from hospital visits and fatal events that were not included in CPRD GOLD to be included in the analyses. Individuals included in this study were adults (aged 18 years or older) with at least one height measurement and one weight measurement available for calculating BMI during the index period (January 2000–December 2010) and registered in the database for at least 3 years before the date of BMI measurement (baseline BMI) within the index period (Figure 1). If an individual had more than one BMI recorded during the index period, the earliest following 3 years of enrollment in the database was taken as baseline BMI. Individuals included in the study population were followed up for occurrence of relevant outcomes for a median of 11.4 years during the study period (until January 2019; Figure 2 and Table 1).

**TABLE 1:** Baseline characteristics of the study population (*n* = 2,924,952) by BMI group

	All individuals	BMI group, kg/m^2^				
		18.5–24.9 (reference group)	25.0–29.9 Overweight	30.0–34.9 Obesity I	35.0–39.9 Obesity II	40.0–45.0 Obesity III
**Number of individuals**	2,924,952	1,099,106	1,074,953	507,425	176,237	67,231
**Sex, number of women (%)**	1,672,338 (57.2)	710,121 (64.6)	534,496 (49.7)	269,204 (53.1)	110,876 (62.9)	47,641 (70.9)
**Smoking, number who ever smoked (%)**	1,455,484 (49.8)	541,522 (49.3)	539,516 (50.2)	256,621 (50.6)	86,026 (48.8)	31,799 (47.3)
**Median age, years (IQR)**	51 (37–64)	47 (33–63)	54 (40–66)	52 (40–64)	49 (38–61)	47 (37–58)
**Median weight, kg (IQR)**	75.0 (64.4–87.0)	63.0 (57.0–69.0)	77.0 (70.0–85.0)	90.0 (82.1–98.5)	101.6 (93.4–111.6)	114.3 (105.0–125.0)
**Median follow‐up, years (IQR)**	11.4 (6.8–14.8)	10.9 (5.8–14.5)	11.6 (7.4–14.9)	11.7 (7.9–14.9)	11.6 (7.9–15.1)	11.5 (7.8–14.9)

Abbreviations: BMI, body mass index; IQR, interquartile range.

The study population was stratified into five groups based on baseline BMI. Individuals with a BMI of 18.5–24.9 kg/m^2^ were considered normal weight and used as the reference group in the data analyses. The other groups were BMI 25.0–29.9 kg/m^2^ (overweight), BMI 30.0–34.9 kg/m^2^ (obesity I), BMI 35.0–39.9 kg/m^2^ (obesity II), and BMI 40.0–45.0 kg/m^2^ (obesity III). Individuals with a BMI less than 18.5 kg/m^2^ were not included because they were considered to be outside the scope of a study studying the impact of overweight and obesity. Furthermore, variation in the underlying reasons for low weight, such as eating disorders or cancer, was considered to limit the validity of potential comparisons between this group and other BMI groups. A wider definition of obesity class III, used by the World Health Organization and the UK National Institute for Health and Care Excellence, includes all individuals with a BMI greater than 40 kg/m^2^.^19^, ^20^ In this study, the individuals with a BMI greater than 45.0 kg/m^2^ were more heterogeneous than the other BMI groups because there were a small number of individuals with very high BMI and, therefore, may have skewed the risks and outcomes associated with this BMI group. This limited the validity of potential comparisons with the other BMI categories, each of which covered a 5‐point BMI range; consequently, individuals with a BMI greater than 45.0 kg/m^2^ were excluded from the study.

### 2.2 Exposure and outcomes

The large number of health conditions known or suspected to be associated with obesity means that the precise impact of obesity itself can be difficult to quantify fully. On the basis of a comprehensive report by the World Health Organization, which indicated that diseases across multiple organ systems are linked to obesity,^21^ a broad range of conditions and events were selected for inclusion in analyses, which represent the cardiovascular, metabolic, endocrine, musculoskeletal, respiratory and renal systems.

For ease of reporting, the 12 obesity‐related conditions and events in the analysis were grouped into five categories. The first category comprised the high‐prevalence conditions T2D, hypertension and dyslipidemia. The second category comprised other noncardiovascular conditions: asthma, sleep apnea, and osteoarthritis, whereas the third category comprised cardiovascular–metabolic conditions (heart failure, chronic kidney disease [CKD] and atrial fibrillation). The fourth category was acute cardiovascular events, comprising two combined cardiovascular outcomes: unstable angina/MI and transient ischemic attack (TIA)/stroke. Finally, all‐cause mortality was captured by extracting death dates from CPRD GOLD.

Conditions and events were identified by the presence of Read codes in CPRD GOLD and International Classification of Diseases, 10th revision codes in Hospital Episode Statistics and Office for National Statistics data (Tables S1 and S2), with the date of earliest diagnosis considered to be the event date (incident diagnosis). For hypertension and dyslipidemia, the event date was either the prescription date of anti‐hypertensive or lipid‐lowering medication, respectively, or the diagnosis date, whichever occurred first. The number of days from baseline to the diagnosis date was used as the time to event. Follow‐up of each individual for a specific condition ended at the date of first relevant diagnosis, death, transfer‐out date or study end (1 January 2019), whichever occurred first.

Diagnoses that occurred before the start of follow‐up were also captured in this study and were considered to constitute baseline comorbidities. When generating the model for any given condition, individuals with the corresponding baseline comorbidity were excluded from the analysis. However, individuals with both baseline and incident diagnoses of acute cardiovascular events were eligible for inclusion in survival analyses, because unstable angina/MI and TIA/stroke were modeled as potential recurring events rather than as one‐time chronic diagnoses.

### 2.3 Statistical analyses

Descriptive data for baseline characteristics were presented as median with interquartile range for continuous variables and as number and proportion (%) for categorical variables. The prevalence of each comorbidity at baseline was calculated based on corresponding diagnoses or events recorded before the baseline date. These data were presented for the full cohort and for strata based on BMI group.

Cox proportional hazard models with age as the underlying timescale were used to estimate the associations between BMI and each obesity‐related condition or event. A separate model was developed for each condition/event and used to calculate the hazard ratio (HR) and 95% confidence interval (CI) for each BMI group relative to the reference group. BMI was described as a categorical variable, with the normal weight group (BMI 18.5–24.9 kg/m^2^) as the reference category in the model. The analyses were adjusted for sex and smoking status, which were encoded as categorical variables; smoking status was coded as either “ever” or “never” based on any recorded smoking status before the baseline date.

In addition to the main analyses, supplementary analyses were carried out to assess the impact of highly prevalent comorbidities (T2D, hypertension, and dyslipidemia) at baseline and any prevalent cardiovascular event (unstable angina, MI, TIA, or stroke) on the outcomes of interest. Cox proportional hazard models with age as the underlying timescale and adjusted for BMI group, sex, and smoking status were used, and the four comorbidities were encoded as categorical variables indicating presence/absence of the comorbidity at baseline. All statistical analyses were carried out using the R environment for statistical computing and visualization (version 3.4.4).

### 2.4 Ethical approval and use of data

The CPRD is a real‐world research service supporting retrospective and prospective public health and clinical studies and is jointly sponsored by the Medicines and Healthcare Products Regulatory Agency and the National Institute for Health Research. The protocol for this study using secondary data was approved by the CPRD Independent Scientific Advisory Committee (protocol number 18_147), in accordance with the Declaration of Helsinki. Data were used in accordance with the terms agreed to upon their receipt. CPRD collects de‐identified patient data from a network of GP practices across the United Kingdom, and this study is therefore based on pseudonymized data from the CPRD.

## 3 RESULTS

### 3.1 Baseline characteristics

**FIGURE 3:**
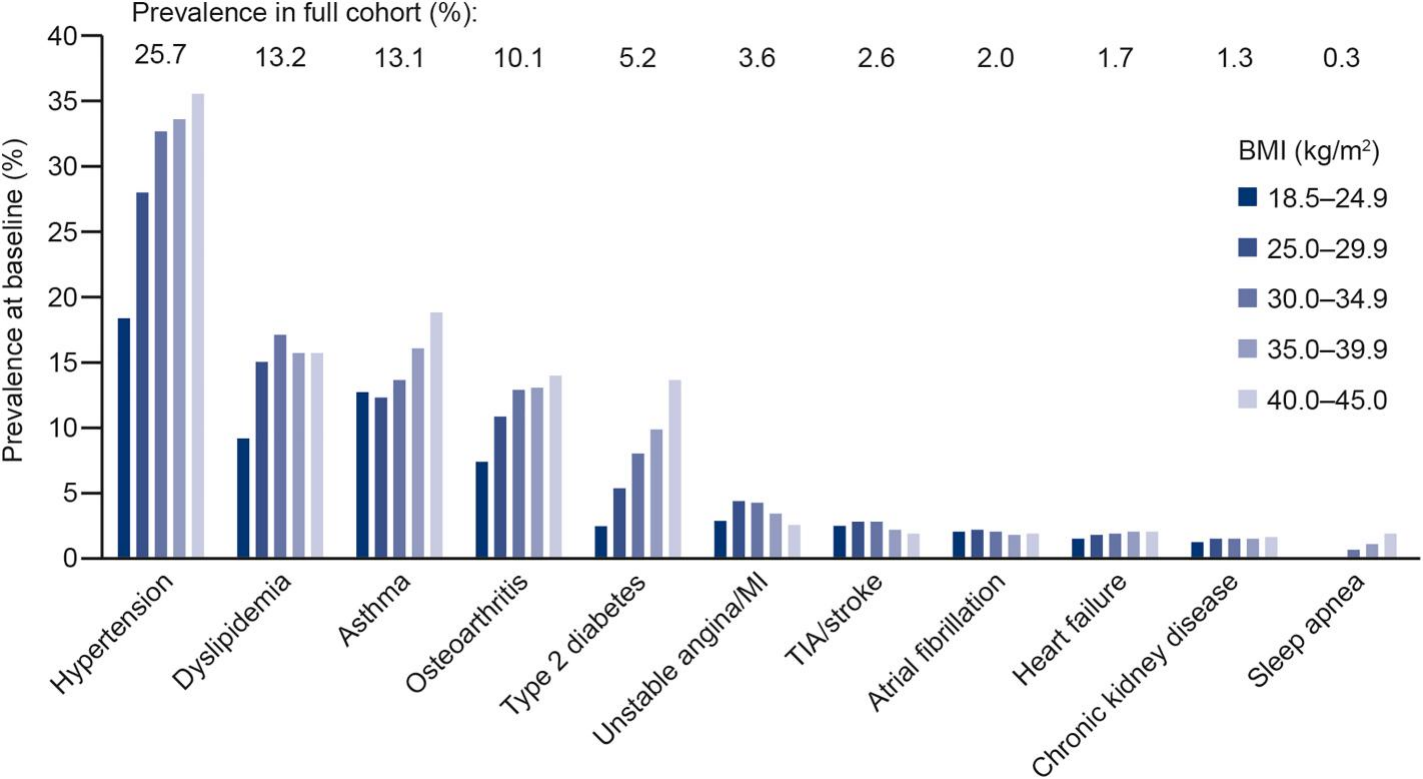
Prevalence of comorbidities in the study population (*n* = 2,924,952) at baseline. Bars indicate separate BMI groups and labels show prevalence in full study population. MI, myocardial infarction; TIA, transient ischemic attack

The study population included a total of 2,924,952 individuals. The median age of the study population at baseline was 51 years (interquartile range [IQR]: 37–64), and the median BMI was 26.5 kg/m^2^ (IQR: 23.5–30.1). In total, 57% of the study population were women. Individuals in the overweight and obesity I groups had a greater average age and a higher proportion of men than the total study population, whereas those in the normal weight, obesity II and obesity III groups were younger on average and contained a higher proportion of women. Baseline characteristics and prevalence of comorbidities at baseline are shown in Table 1 and Figure 3, respectively.

In the total study population, the most common baseline comorbidities were hypertension (present in 25.7% of the population), dyslipidemia (13.2%), asthma (13.1%), osteoarthritis (10.1%), and T2D (5.2%). Across all BMI groups, the prevalence of these five comorbidities was typically higher in individuals with higher BMI. However, unstable angina/MI, TIA/stroke and atrial fibrillation events had similar or lower prevalence at baseline in the obesity II and obesity III groups compared with lower BMI groups.

### 3.2 BMI and risk of obesity‐related outcomes

**FIGURE 4:**
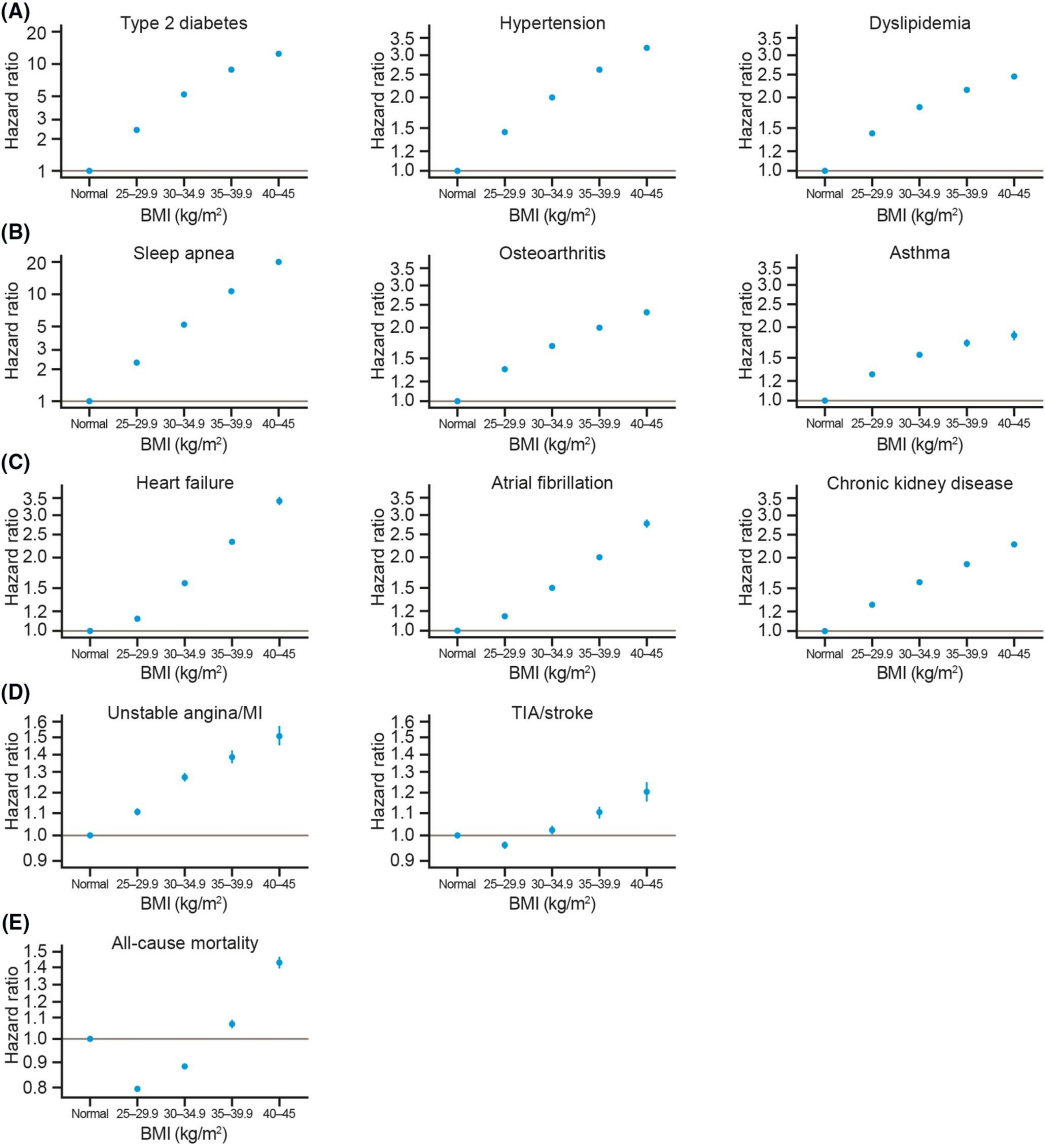
Risk of (A) high‐prevalence conditions, (B) noncardiovascular conditions, (C) cardiovascular–metabolic conditions, (D) cardiovascular events, and (E) all‐cause mortality, by BMI group. Data shown are hazard ratios with 95% confidence intervals for each BMI group relative to the reference group (normal weight), derived from Cox proportional hazard models adjusted for age, sex, and smoking status. Note that *y*‐axis scales differ between conditions. BMI, body mass index; MI, myocardial infarction; TIA, transient ischemic attack

Figure 4 shows the risk of (A) high‐prevalence conditions, (B) noncardiovascular conditions, (C) cardiovascular–metabolic conditions, (D) cardiovascular events, and (E) all‐cause mortality for all BMI groups during the follow‐up period. Table S3 presents the corresponding HRs and 95% CIs.

Compared with the reference group (18.5–24.9 kg/m^2^), the highest BMI group (40.0–45.0 kg/m^2^) had a higher risk of all conditions and events. Individuals with a BMI of 40.0–45.0 kg/m^2^ were at particularly high risk of sleep apnea (HR [95% CI]: 19.8 [18.9–20.8]) and T2D (12.4 [12.1–12.7]). These individuals also had a more than threefold higher risk of heart failure (3.46 [3.35–3.57]) and hypertension (3.21 [3.15–3.26]) than individuals in the reference group. HRs for all other outcomes examined were in the range of 1.2–2.8 for the BMI 40.0–45.0 kg/m^2^ group compared with the reference group.

In general, there was a stepwise increase in risk of high‐prevalence conditions, noncardiovascular conditions, cardiovascular–metabolic conditions and unstable angina/MI with increasing BMI group; however, individuals with a BMI of 25.0–29.9 kg/m^2^ had a slightly lower risk of TIA/stroke than those in the reference group (HR [95% CI]: 0.96 [0.95–0.97]). This pattern was also apparent in the analysis for all‐cause mortality, in both the BMI 25.0–29.9 kg/m^2^ and the BMI 30–34.9 kg/m^2^ groups (HRs [95% CIs]: 0.80 [0.79–0.81] and 0.88 [0.88–0.89], respectively).

Smoking was associated with a higher risk of all conditions and events studied, in particular unstable angina/MI (HR [95% CI]: 1.54 [1.52–1.56]) and mortality (1.57 [1.56–1.58]). Male sex was a risk factor for all conditions and events with the exception of osteoarthritis (0.69 [0.68–0.69]), asthma (0.72 [0.71–0.73]), and CKD (0.91 [0.91–0.92]), which were more common in women (Table S3).

### 3.3 Contribution of baseline comorbidities to risk of obesity‐related outcomes

**FIGURE 5:**
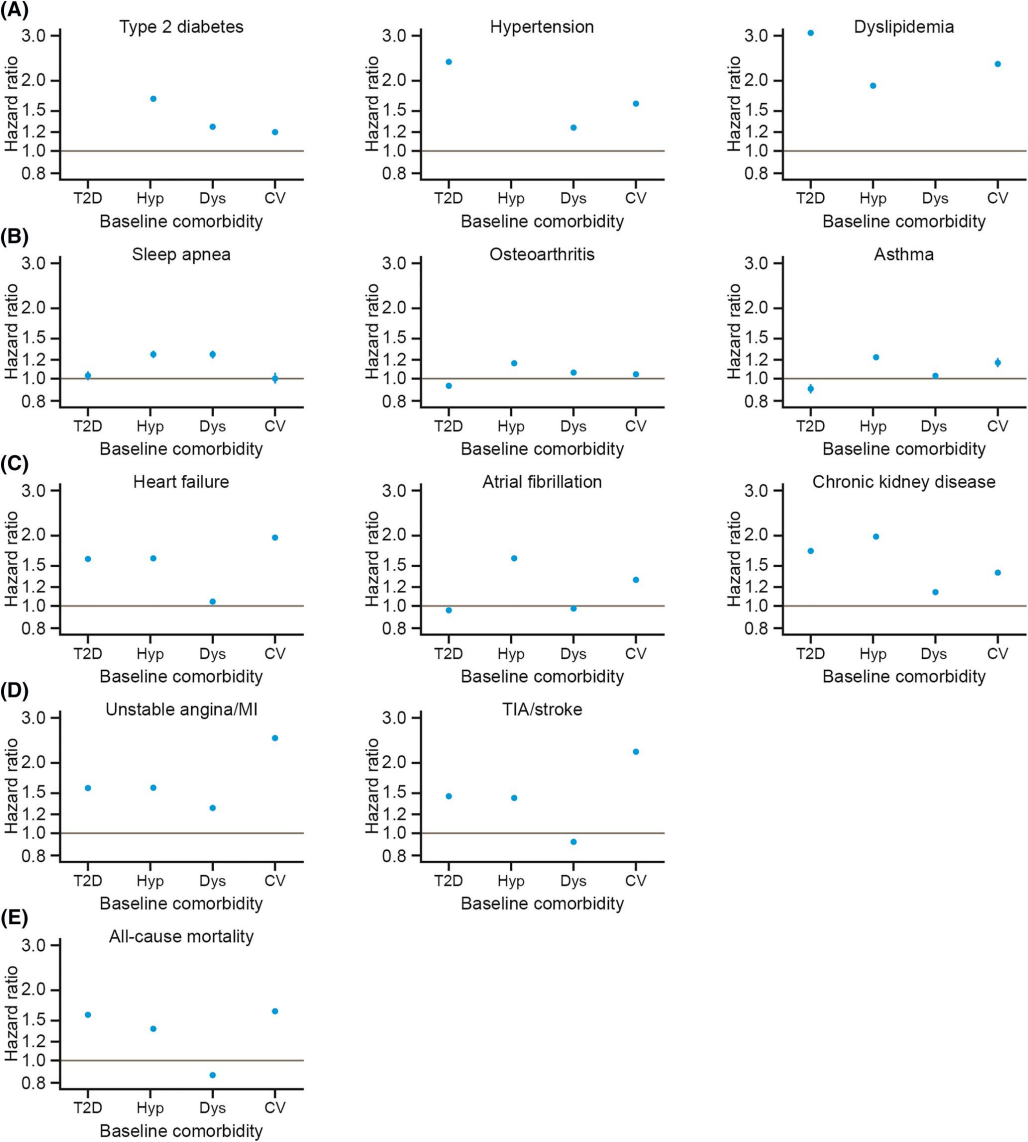
Risk of (A) high‐prevalence conditions, (B) noncardiovascular conditions, (C) cardiovascular–metabolic conditions, (D) cardiovascular events and (E) all‐cause mortality, by presence of high‐prevalence baseline comorbidities. Data shown are hazard ratios with 95% confidence intervals for each high‐prevalence baseline comorbidity (type 2 diabetes, hypertension, dyslipidemia, and cardiovascular disease) relative to the absence of that comorbidity, derived from Cox proportional hazard models adjusted for age, sex, smoking status, and presence of high‐prevalence baseline comorbidities. BMI, body mass index; CV, history of cardiovascular event(s); Dys, dyslipidemia; Hyp, hypertension; MI, myocardial infarction; T2D, type 2 diabetes; TIA, transient ischemic attack

When the associations between high‐prevalence baseline comorbidities, cardiovascular disease history and incident diagnoses occurring during the study period were examined, individuals with a previous diagnosis of T2D, hypertension, or dyslipidemia, or a history of cardiovascular events, were at higher risk of experiencing particular conditions than those without these baseline comorbidities (Figure 5 and Table S4).

T2D at baseline was associated with a more than threefold higher risk of being diagnosed with dyslipidemia (HR [95% CI]: 3.12 [3.10–3.15]) and a more than doubled risk of being diagnosed with hypertension (2.34 [2.32–2.37]). Individuals who had hypertension at baseline had nearly twice the risk of being diagnosed with CKD (1.93 [1.92–1.95]) or dyslipidemia (1.87 [1.86–1.88]), and also had an increased risk of T2D diagnosis (1.68 [1.67–1.70]), compared with those who were not diagnosed with hypertension before the start of the study period. The baseline presence of dyslipidemia was associated with a comparatively higher risk of a diagnosis of T2D (1.28 [1.26–1.30]), sleep apnea (1.26 [1.22–1.30]), or unstable angina/MI (1.26 [1.25–1.28]) during the study period.

Individuals with a history of any cardiovascular event had a more than twofold higher risk of experiencing an unstable angina/MI event (2.47 [2.43–2.51]), a dyslipidemia diagnosis (2.31 [2.28–2.33]), or a TIA/stroke event (2.17 [2.14–2.20]), and a nearly twofold higher risk of heart failure (1.91 [1.88–1.94]) during the study period, compared with those who had never experienced a cardiovascular event.

## 4 DISCUSSION

This study substantiates evidence of a link between high BMI and 12 serious health conditions and events in a cohort of over 2.9 million adults, representative of the UK population. Individuals in the highest BMI group had a substantially higher risk of all outcomes examined, with a 20‐times higher risk for sleep apnea, a 12‐times higher risk for T2D, and a threefold higher risk of experiencing certain cardiovascular conditions, compared with individuals of normal weight. The risk for most conditions was generally greater in higher BMI groups, relative to groups with lower BMI. These findings are in line with previous studies showing a positive correlation between BMI and risk of these chronic conditions and cardiovascular events.^5^, ^6^, ^7^ Individuals' sex and smoking status affected the occurrence of health outcomes; furthermore, individuals with T2D, hypertension, or dyslipidemia at baseline had an increased risk for developing another one of these three conditions in the future. There was also an association between baseline dyslipidemia or a history of cardiovascular events and the occurrence of these conditions during the study period.

In this analysis, individuals in the overweight group had a higher risk of most outcomes than individuals in the reference group with healthy weight; however, this was not the case for TIA/stroke and all‐cause mortality, for which individuals in the overweight and obesity I groups had a risk of experiencing these outcomes that was similar to or lower than that for individuals with normal weight. These results for all‐cause mortality are in line with the findings of several previous meta‐analyses, which reported a J‐shaped distribution for mortality plotted against BMI.^9^, ^10^, ^16^, ^22^, ^23^, ^24^ Furthermore, an analysis of the Framingham Heart Study found lower mortality following ischemic stroke in individuals with BMI 25–30 kg/m^2^ compared with those who had normal weight.^25^ A possible explanation for the mortality findings is that illness can cause weight loss, leading to reverse causation bias: namely, conditions that carry an imminent risk of mortality can cause reductions in BMI, rather than BMI reductions causing mortality. This can lead to an underestimation of the mortality risks associated with overweight or obesity.^9^, ^26^ To mitigate possible underestimation of mortality risks, a study design with long follow‐up was used,^9^ and adjustment were made for known potential confounding factors including age,^23^ sex,^23^ and smoking history.^9^, ^24^ However, residual confounding may still be present.

In general, the prevalence of baseline comorbidities in the study cohort increased with higher BMI, with the exceptions of unstable angina/MI, TIA/stroke, and atrial fibrillation, which had similar or lower prevalence in the obesity II and obesity III groups relative to other BMI groups. This may be due to the disparities in median age and sex across the BMI groups, which resulted in a greater proportion of women and lower average age in the higher BMI groups than in the other groups. This pattern may be attributable to survival bias: older individuals with more severe obesity are likely to have more cardiovascular comorbidities and consequently higher mortality; therefore, surviving individuals in these groups may have a disproportionately low rate of certain comorbidities.

A major strength of this study is the utilization of a large, UK‐representative primary care database, which allowed us to examine a broad range of conditions in a single study population and to compare the relative magnitude of risks between outcomes. Furthermore, the large size of the study population, the long follow‐up period, and the use of data from real‐world clinical practice mean that the results are robust and highly generalizable. Including individuals on the basis of a single eligible BMI measurement during the index period greatly increased the available sample size, meaning that the analyses had strong statistical power to estimate the relationship between BMI and future disease risk with a high degree of confidence. However, this study design meant that BMI was not tracked over time, and therefore a more precise relationship between individual BMI changes and occurrence of obesity‐related conditions cannot be inferred from the data.

Observational studies such as the present study can be subject to confounding and other biases. Potential confounders, particularly details about individuals' lifestyles and certain demographic factors, such as ethnicity, are unlikely to be fully captured in primary care records and therefore are not taken into account in the analyses. Although diagnostic codes and prescriptions data are an acceptable means of detecting disease incidence in such data sources, disparities in how certain conditions are detected, formally diagnosed and treated mean that this method can lead to both over‐ and underpredictions of outcome risks. This can also create bias when assessing associations between conditions; for example, the prescription of statins to treat dyslipidemia may increase T2D risk.^27^ Finally, although BMI is a reliable screening tool for overweight and obesity, and is the best means of defining these conditions when using data sources in which only height and weight data are routinely collected, it should not be used in isolation when other data are available. Waist circumference and body fat percentage are also valuable indicators of obesity or obesity‐related risks and should be taken into consideration in clinical practice.^28^, ^29^, ^30^

Another limitation of the study was the requirement to exclude individuals with a BMI greater than 45 kg/m^2^; however, it is likely that estimation of outcome risks for this group would have been skewed by a number of individuals with very high BMI. Additionally, the inclusion criterion requiring at least 3 years' registration in CPRD to capture adequate historical data meant that a large number of individuals were automatically excluded from the analysis, creating a potential for bias. Furthermore, there were differences between sample sizes in the study, which means that the large disparities in risks between the highest BMI group (*n* = 67,231) and the reference group (*n* = 1,099,106) should be treated with a degree of caution. It should also be noted that individuals with higher BMI and/or more conditions of interest are disproportionately likely to have contact with primary care services, meaning that comparatively fewer healthcare data are available for individuals of normal weight. Therefore, this study could conceivably have been biased toward detecting individuals with higher BMI. However, reporting of BMI in CPRD increased during the study period, and from 2005 to 2011, 77% of included individuals had a previous BMI measurement,^31^ which partly mitigates this risk of bias.

The results of this study highlight the clear association between BMI and the risk of future health outcomes and provide a valuable basis for further research into obesity. Subsequent analyses are needed to quantify the contribution of other relevant risk factors, such as height, and, with modifications to our study design, the CPRD could also be used to examine the impact of obesity on mental health in the same population. To show associations between changes in BMI over time and obesity‐related complications, a different, longitudinal study design would be required; indeed, this has been carried out in a subsequent study using the CPRD.

Recent studies using the CPRD have also highlighted the additional resource use incurred in populations who have T2D or cardiovascular disease in addition to obesity.^32^, ^33^ Although a sizable proportion of obesity‐related costs and resource use are known to be contributed by complications of the disease,^34^, ^35^ there is considerable variation in which conditions are included in overall cost estimation for obesity.^36^ Therefore, as well as demonstrating the cost benefits to be gained by a reduction in obesity rates,^37^ data quantifying the impact of BMI on the risk of chronic conditions are needed to generate reliable inputs for economic evaluations of obesity.

Despite public health programs and management strategies, obesity prevalence remains high in many nations.^3^ The clinical, societal, and economic impacts of obesity have been brought into sharper focus by the coronavirus disease 2019 (COVID‐19) pandemic. International data indicate that obesity is a risk factor for hospitalization and mortality associated with COVID‐19,^38^ and preliminary studies in the UK populations have suggested that not only is higher BMI positively correlated with COVID‐19 severity and mortality, but other conditions that commonly occur as complications of obesity, such as T2D and hypertension, are also risk factors for poor outcomes related to COVID‐19.^39^, ^40^, ^41^ These findings have particularly severe implications in the United Kingdom, which both have some of the highest rates of overweight and obesity worldwide^3^ and, at the time of writing, have one of the highest numbers of COVID‐19‐related deaths per million people.^42^ Furthermore, as the number of COVID‐19 cases increases, individuals with chronic health conditions are also likely to experience disruption to routine care due to increased pressure on healthcare systems.^43^ As a consequence of the pandemic, public health and clinical strategies to reduce obesity rates have been announced as a major priority in the United Kingdom.^44^, ^45^ The results of the present study illustrate how vitally this is needed for individuals, healthcare systems and society.

The results of this study indicate that higher BMI is associated with increased risks for a range of serious health conditions. This demonstrates the requirement for effective treatments for individuals living with obesity, as well as wider public health and health educational programs to slow and limit the development of the disease on a population level.^46^ Only via such measures can the considerable societal costs of obesity and its related conditions be mitigated, and its impact on healthcare systems^37^ managed.

## CONFLICTS OF INTEREST

Christiane L. Haase, Kirsten T. Eriksen, Sandra Lopes, Altynai Satylganova and Volker Schnecke are employees of Novo Nordisk A/S. Altynai Satylganova and Sandra Lopes are also shareholders of Novo Nordisk A/S. Phil McEwan is an employee of Health Economics and Outcomes Research Ltd. Phil McEwan did not receive funding for this collaboration. HEOR Ltd have received funding from Novo Nordisk A/S for work conducted on previous studies.

## Supporting information

Supporting Information

## References

[1] World Health Organization . Overweight and Obesity. 2018. http://www.who.int/news-room/fact-sheets/detail/obesity-and-overweight. Accessed 10 June 2019.

[2] National Health Service . Statistics on Obesity, Physical Activity and Diet, England, 2019. Part 3: Adult Overweight and Obesity. 2019. https://digital.nhs.uk/data-and-information/publications/statistical/statistics-on-obesity-physical-activity-and-diet/statistics-on-obesity-physical-activity-and-diet-england-2019/part-3-adult-obesity. Accessed 10 June 2019.

[3] Organisation for Economic Co‐operation and Development (OECD) . OECD Data. Overweight or obese population. 2019. https://data.oecd.org/healthrisk/overweight-or-obese-population.htm. Accessed 26 September 2020.

[4] Organisation for Economic Co‐operation and Development (OECD) . OECD Obesity Update. 2017. Accessed 25 September 2020.

[5] Li C , Ford ES , Zhao G , Croft JB , Balluz LS , Mokdad AH . Prevalence of self‐reported clinically diagnosed sleep apnea according to obesity status in men and women: National Health and Nutrition Examination Survey, 2005‐2006. Prev Med. 2010;51:18‐23. 20381517 10.1016/j.ypmed.2010.03.016

[6] Must A , Spadano J , Coakley EH , Field AE , Colditz G , Dietz WH . The disease burden associated with overweight and obesity. JAMA. 1999;282:1523‐1529. 10546691 10.1001/jama.282.16.1523

[7] Khan SS , Ning H , Wilkins JT , et al. Association of body mass index with lifetime risk of cardiovascular disease and compression of morbidity. JAMA Cardiol. 2018;3:280‐287. 29490333 10.1001/jamacardio.2018.0022 PMC5875319

[8] G. B. D. Obesity Collaborators , Afshin A , Forouzanfar MH , et al. Health effects of overweight and obesity in 195 countries over 25 years. N Engl J Med. 2017;377:13‐27. 28604169 10.1056/NEJMoa1614362 PMC5477817

[9] Aune D , Sen A , Prasad M , et al. BMI and all‐cause mortality: systematic review and non‐linear dose‐response meta‐analysis of 230 cohort studies with 3.74 million deaths among 30.3 million participants. BMJ. 2016;353:i2156. 27146380 10.1136/bmj.i2156 PMC4856854

[10] Flegal KM , Kit BK , Orpana H , Graubard BI . Association of all‐cause mortality with overweight and obesity using standard body mass index categories: a systematic review and meta‐analysis. JAMA. 2013;309:71‐82. 23280227 10.1001/jama.2012.113905 PMC4855514

[11] Public Health England . Health matters: obesity and the food environment. 2017. https://www.gov.uk/government/publications/health-matters-obesity-and-the-food-environment/health-matters-obesity-and-the-food-environment--2. Accessed 14 October 2019.

[12] Wang YC , McPherson K , Marsh T , Gortmaker SL , Brown M . Health and economic burden of the projected obesity trends in the USA and the UK. Lancet. 2011;378:815‐825. 21872750 10.1016/S0140-6736(11)60814-3

[13] Counterweight Project Team . The impact of obesity on drug prescribing in primary care. Br J Gen Pract. 2005;55:743‐749. 16212848 PMC1562331

[14] Hoque ME , Mannan M , Long KZ , Al Mamun A . Economic burden of underweight and overweight among adults in the Asia‐Pacific region: a systematic review. Trop Med Int Health. 2016;21:458‐469. 26892222 10.1111/tmi.12679

[15] CPRD . CPRD Bibliography. 2020. https://www.cprd.com/bibliography. Accessed 27 September 2020.

[16] Bhaskaran K , Dos‐Santos‐Silva I , Leon DA , Douglas IJ , Smeeth L . Association of BMI with overall and cause‐specific mortality: a population‐based cohort study of 3.6 million adults in the UK. Lancet Diabetes Endocrinol. 2018;6:944‐953. 30389323 10.1016/S2213-8587(18)30288-2 PMC6249991

[17] CPRD . Primary Care Data for Public Health Research. 2020. https://cprd.com/primary-care. Accessed 20 April 2020.

[18] Herrett E , Gallagher AM , Bhaskaran K , et al. Data resource profile: clinical practice research datalink (CPRD). Int J Epidemiol. 2015;44:827‐836. 26050254 10.1093/ije/dyv098 PMC4521131

[19] National Institute for Health and Care Excellence . Identifying and Assessing People Who Are Overweight or Obese. Based on Obesity: Identification, Assessment and Management. 2014. NICE guideline CG189. https://pathways.nice.org.uk/pathways/obesity?fno=1#path=view%3A/pathways/obesity/identifying-and-assessing-people-who-are-overweight-or-obese.xml&content=view-node%3Anodes-bmi-adult. Accessed 25 September 2019.

[20] World Health Organization Regional Office for Europe . Body mass index ‐ BMI. 2019. http://www.euro.who.int/en/health-topics/disease-prevention/nutrition/a-healthy-lifestyle/body-mass-index-bmi. Accessed 15 October 2019.

[21] World Health Organization . WHO technical report series. Obesity: preventing and managing the global epidemic Report of a WHO Consultation (WHO Technical Report Series 894). 2000. https://www.who.int/nutrition/publications/obesity/WHO_TRS_894/en/. Accessed 14 October 2019. 11234459

[22] Global BMIMC , Di Angelantonio E , Bhupathiraju SN , et al. Body‐mass index and all‐cause mortality: individual‐participant‐data meta‐analysis of 239 prospective studies in four continents. Lancet. 2016;388:776‐786. 27423262 10.1016/S0140-6736(16)30175-1 PMC4995441

[23] Peter RS , Mayer B , Concin H , Nagel G . The effect of age on the shape of the BMI‐mortality relation and BMI associated with minimum all‐cause mortality in a large Austrian cohort. Int J Obes. 2015;39:530‐534. 10.1038/ijo.2014.168 25214148

[24] Sun YQ , Burgess S , Staley JR , et al. Body mass index and all‐cause mortality in HUNT and UK Biobank studies: linear and non‐linear mendelian randomisation analyses. BMJ. 2019;364:l1042. 30957776 10.1136/bmj.l1042 PMC6434515

[25] Aparicio HJ , Himali JJ , Beiser AS , et al. Overweight, obesity, and survival after stroke in the Framingham heart study. J Am Heart Assoc. 2017;6:e004721. 28647687 10.1161/JAHA.116.004721 PMC5669145

[26] Yu E , Ley SH , Manson JE , et al. Weight history and all‐cause and cause‐specific mortality in three prospective cohort studies. Ann Intern Med. 2017;166:613‐620. 28384755 10.7326/M16-1390 PMC5518318

[27] Sattar N , Preiss D , Murray HM , et al. Statins and risk of incident diabetes: a collaborative meta‐analysis of randomised statin trials. Lancet. 2010;375:735‐742. 20167359 10.1016/S0140-6736(09)61965-6

[28] Bouchard C . BMI, fat mass, abdominal adiposity and visceral fat: where is the ‘beef’? Int J Obes. 2007;31:1552‐1553. 10.1038/sj.ijo.0803653 17549092

[29] De Lorenzo A , Bianchi A , Maroni P , et al. Adiposity rather than BMI determines metabolic risk. Int J Cardiol. 2013;166:111‐117. 22088224 10.1016/j.ijcard.2011.10.006

[30] Rueda‐Clausen CF , Poddar M , Lear SA , Poirier P , Sharma AM . Canadian Adult Obesity Clinical Practice Guidelines: Assessment of People Living with Obesity. Version 1. 2020. https://obesitycanada.ca/guidelines/assessment. Accessed 29 October 2020.

[31] Bhaskaran K , Forbes HJ , Douglas I , Leon DA , Smeeth L . Representativeness and optimal use of body mass index (BMI) in the UK Clinical Practice Research Datalink (CPRD). BMJ Open. 2013;3:e003389. 10.1136/bmjopen-2013-003389 PMC3773634 24038008

[32] le Roux CW , Chubb B , Nortoft E , Borglykke A . Obesity and healthcare resource utilization: results from clinical practice research database (CPRD). Obes Sci Pract. 2018;4:409‐416. 30338111 10.1002/osp4.291 PMC6180713

[33] le Roux CW , Hartvig NV , Haase CL , Nordsborg RB , Olsen AH , Satylganova A . Obesity, cardiovascular risk and healthcare resource utilization in the UK. Eur J Prev Cardiol. 2020. 2047487320925639. 34551077 10.1177/2047487320925639

[34] Kent S , Green J , Reeves G , et al. Hospital costs in relation to body‐mass index in 1.1 million women in England: a prospective cohort study. Lancet Public Health. 2017;2:e214‐e22. 29253487 10.1016/S2468-2667(17)30062-2 PMC6196771

[35] Kent S , Jebb SA , Gray A , et al. Body mass index and use and costs of primary care services among women aged 55‐79 years in England: a cohort and linked data study. Int J Obes. 2019;43:1839‐1848. 10.1038/s41366-018-0288-6 PMC6451629 30568274

[36] Tremmel M , Gerdtham UG , Nilsson PM , Saha S . Economic burden of obesity: a systematic literature review. Int J Environ Res Publ Health. 2017;14. 10.3390/ijerph14040435 PMC5409636 28422077

[37] Cawley J , Meyerhoefer C , Biener A , Hammer M , Wintfeld N . Savings in medical expenditures associated with reductions in body mass index among US Adults with obesity, by diabetes status. Pharmacoeconomics. 2015;33:707‐722. 25381647 10.1007/s40273-014-0230-2 PMC4486410

[38] Popkin BM , Du S , Green WD , et al. Individuals with obesity and COVID‐19: a global perspective on the epidemiology and biological relationships. Obes Rev. 2020;21. 10.1111/obr.13128 PMC7461480 32845580

[39] Docherty AB , Harrison EM , Green CA , et al. Features of 16,749 hospitalised UK patients with COVID‐19 using the ISARIC WHO clinical characterisation protocol. medRxiv. 2020. 10.1101/2020.04.23.20076042 PMC7243036 32444460

[40] Khawaja AP , Warwick AN , Hysi PG , et al. Associations with covid‐19 hospitalisation amongst 406,793 adults: the UK Biobank prospective cohort study. medRxiv. 2020. 10.1101/2020.05.06.20092957

[41] Williamson E , Walker AJ , Bhaskaran KJ , et al. OpenSAFELY: factors associated with COVID‐19‐related hospital death in the linked electronic health records of 17 million adult NHS patients. medRxiv. 2020. 10.1101/2020.05.06.20092999

[42] World Health Organization . WHO coronavirus disease (COVID‐19) dashboard. 2020. https://covid19.who.int/. Accessed 25 September 2020.

[43] Chudasama YV , Gillies CL , Zaccardi F , et al. Impact of COVID‐19 on routine care for chronic diseases: a global survey of views from healthcare professionals. Diabetes Metab Syndr. 2020;14:965‐967. 32604016 10.1016/j.dsx.2020.06.042 PMC7308780

[44] Cuthbertson DJ , Alam U , Tahrani A . COVID‐19 and obesity: an opportunity for change. Ther Adv Endocrinol Metab. 2020;11:2042018820949742. 32913624 10.1177/2042018820949742 PMC7441482

[45] Mason L . Obesity in the age of COVID‐19. Perspect Public Health. 2020;140:247‐248. 32933428 10.1177/1757913920943390

[46] Burguera B , Fitch A , Owens GM , Patel D , San Martin VT . Management of obesity: considerations in managed care medicine. J Manag Care Med. 2018. http://jmcmpub.org/wp-content/uploads/2019/06/ObesitySupplement.pdf

