# Supplementary material for: Body mass index and risk of obesity‐related conditions in a cohort of 2.9 million people: Evidence from a UK primary care database
Source: Obes Sci Pract. 2020 Dec 24;7(2):137–47. doi: 10.1002/osp4.474 (PMC8019280; doi:10.1002/osp4.474)
Supplement: Supplementary file 1 — Supporting Information [file OSP4-7-137-s001.pdf]

# Body mass index and risk of obesity-related conditions in a cohort of 2.9 million people: evidence from a UK primary care database

**Authors**

Christiane L. Haase[1], Kirsten T. Eriksen[1], Sandra Lopes[1], Altynai Satylganova[1], Volker Schnecke[1] and Phil McEwan[2]

**Author affiliations**

[1] Novo Nordisk A/S, Søborg, Denmark

[2] Health Economics and Outcomes Research Ltd, Cardiff, UK

## Supplemental material

Table S1 Read codes used to identify conditions and events in CPRD GOLD

Table S2 ICD-10 codes used to identify conditions and events in HES and ONS data

Table S3 Risks for each condition and event during the follow-up period

Table S4 Risks for each condition and event in supplementary analyses adjusted for baseline comorbidities

**Table S1 Read codes used to identify conditions and events in CPRD GOLD**

| type | medcode | read_code | desc |
|---|---|---|---|
| Atrial fibrillation | 1268 | G573200 | Paroxysmal atrial fibrillation |
| Atrial fibrillation | 1664 | G573000 | Atrial fibrillation |
| Atrial fibrillation | 1757 | G573100 | Atrial flutter |
| Atrial fibrillation | 2212 | G573.00 | Atrial fibrillation and flutter |
| Atrial fibrillation | 23437 | G573z00 | Atrial fibrillation and flutter NOS |
| Atrial fibrillation | 35127 | G573300 | Non-rheumatic atrial fibrillation |
| Atrial fibrillation | 96076 | G573500 | Persistent atrial fibrillation |
| Atrial fibrillation | 96277 | G573400 | Permanent atrial fibrillation |
| Atrial fibrillation | 107472 | G573600 | Paroxysmal atrial flutter |
| Asthma | 78 | H33..00 | Asthma |
| Asthma | 185 | H333.00 | Acute exacerbation of asthma |
| Asthma | 232 | H33z100 | Asthma attack |
| Asthma | 233 | H33z011 | Severe asthma attack |
| Asthma | 1208 | H330.12 | Childhood asthma |
| Asthma | 1555 | H33..11 | Bronchial asthma |
| Asthma | 2290 | H330.11 | Allergic asthma |
| Asthma | 3018 | 663V100 | Mild asthma |
| Asthma | 3366 | 663V300 | Severe asthma |
| Asthma | 3458 | 663V000 | Occasional asthma |
| Asthma | 3665 | H331.11 | Late onset asthma |
| Asthma | 4442 | H33z.00 | Asthma unspecified |
| Asthma | 4606 | H33zz11 | Exercise induced asthma |
| Asthma | 4892 | H33z000 | Status asthmaticus NOS |
| Asthma | 5267 | H331.00 | Intrinsic asthma |
| Asthma | 5627 | H330011 | Hay fever with asthma |
| Asthma | 5798 | H312000 | Chronic asthmatic bronchitis |
| Asthma | 5867 | 173A.00 | Exercise induced asthma |
| Asthma | 6707 | H330111 | Extrinsic asthma with asthma attack |
| Asthma | 7146 | H330.00 | Extrinsic (atopic) asthma |
| Asthma | 7731 | H330.14 | Pollen asthma |
| Asthma | 8335 | H33z111 | Asthma attack NOS |
| Asthma | 10487 | 663j.00 | Asthma - currently active |
| Asthma | 11370 | 1O2..00 | Asthma confirmed |
| Asthma | 12987 | H33z200 | Late-onset asthma |

| | | | |
|---|---|---|---|
| Asthma | 13065 | 663V200 | Moderate asthma |
| Asthma | 14777 | H330000 | Extrinsic asthma without status asthmaticus |
| Asthma | 15248 | H330.13 | Hay fever with asthma |
| Asthma | 16070 | H33zz00 | Asthma NOS |
| Asthma | 18207 | H33zz13 | Allergic bronchitis NEC |
| Asthma | 18323 | H331111 | Intrinsic asthma with asthma attack |
| Asthma | 21232 | H33zz12 | Allergic asthma NEC |
| Asthma | 22752 | 173c.00 | Occupational asthma |
| Asthma | 25796 | H332.00 | Mixed asthma |
| Asthma | 27926 | H330100 | Extrinsic asthma with status asthmaticus |
| Asthma | 29325 | H331000 | Intrinsic asthma without status asthmaticus |
| Asthma | 32727 | H33z.11 | Hyperreactive airways disease |
| Asthma | 39478 | H35y700 | Wood asthma |
| Asthma | 40823 | H334.00 | Brittle asthma |
| Asthma | 41017 | 1780 | Aspirin induced asthma |
| Asthma | 45073 | H331z00 | Intrinsic asthma NOS |
| Asthma | 45782 | H330z00 | Extrinsic asthma NOS |
| Asthma | 47684 | H47y000 | Detergent asthma |
| Asthma | 58196 | H331100 | Intrinsic asthma with status asthmaticus |
| Asthma | 73522 | 173d.00 | Work aggravated asthma |
| CKD | 512 | K05..00 | Chronic renal failure |
| CKD | 1803 | K011.00 | Nephrotic syndrome with membranous glomerulonephritis |
| CKD | 2471 | K01x100 | Nephrotic syndrome in diabetes mellitus |
| CKD | 2939 | K100600 | Calculous pyelonephritis |
| CKD | 2999 | K01..00 | Nephrotic syndrome |
| CKD | 4654 | K100.00 | Chronic pyelonephritis |
| CKD | 4669 | K02y200 | Chronic focal glomerulonephritis |
| CKD | 5911 | ZV42000 | [V]Kidney transplanted |
| CKD | 6712 | K050.00 | End stage renal failure |
| CKD | 7804 | K02..00 | Chronic glomerulonephritis |
| CKD | 8330 | K0D..00 | End-stage renal disease |
| CKD | 9840 | K010.00 | Nephrotic syndrome with proliferative glomerulonephritis |
| CKD | 10081 | K05..11 | Chronic uraemia |
| CKD | 10647 | K02..11 | Nephritis - chronic |
| CKD | 10809 | K021.00 | Chronic membranous glomerulonephritis |
| CKD | 11553 | SP08300 | Kidney transplant failure and rejection |

| | | | |
|---|---|---|---|
| CKD | 11875 | K02..12 | Nephropathy - chronic |
| CKD | 12479 | 1Z13.00 | Chronic kidney disease stage 4 |
| CKD | 12566 | 1Z12.00 | Chronic kidney disease stage 3 |
| CKD | 12585 | 1Z14.00 | Chronic kidney disease stage 5 |
| CKD | 15097 | K02z.00 | Chronic glomerulonephritis NOS |
| CKD | 17253 | 8L50.00 | Renal transplant planned |
| CKD | 17365 | K01B.00 | Nephrotic syndrome, diffuse crescentic glomerulonephritis |
| CKD | 18774 | TB00111 | Renal transplant with complication, without blame |
| CKD | 19316 | K016.00 | Nephrotic syndrome, diffuse membranous glomerulonephritis |
| CKD | 21158 | K100200 | Chronic pyelitis |
| CKD | 21947 | K017.00 | Nephrotic syn difus mesangial prolifertiv glomerulonephritis |
| CKD | 21989 | K019.00 | Nephrotic syn,diffuse mesangiocapillary glomerulonephritis |
| CKD | 22205 | K01x411 | Lupus nephritis |
| CKD | 22852 | K015.00 | Nephrotic syndrome, focal and segmental glomerular lesions |
| CKD | 23913 | K014.00 | Nephrotic syndrome, minor glomerular abnormality |
| CKD | 25055 | K100300 | Chronic pyonephrosis |
| CKD | 26862 | 7B06300 | Exploration of renal transplant |
| CKD | 27427 | K01z.00 | Nephrotic syndrome NOS |
| CKD | 29634 | K013.00 | Nephrotic syndrome with minimal change glomerulonephritis |
| CKD | 34998 | K020.00 | Chronic proliferative glomerulonephritis |
| CKD | 35360 | K100400 | Nonobstructive reflux-associated chronic pyelonephritis |
| CKD | 40349 | K013.11 | Lipoid nephrosis |
| CKD | 45499 | K01x111 | Kimmelstiel - Wilson disease |
| CKD | 47672 | K01x400 | Nephrotic syndrome in systemic lupus erythematosus |
| CKD | 47922 | K01x000 | Nephrotic syndrome in amyloidosis |
| CKD | 48111 | K100z00 | Chronic pyelonephritis NOS |
| CKD | 48855 | K100500 | Chronic obstructive pyelonephritis |
| CKD | 50472 | K018.00 | Nephrotic syn,difus endocapilary proliftv glomerulonephritis |
| CKD | 53852 | K05..12 | End stage renal failure |
| CKD | 54990 | TB00100 | Kidney transplant with complication, without blame |
| CKD | 56987 | K01A.00 | Nephrotic syndrome, dense deposit disease |
| CKD | 57568 | K100100 | Chronic pyelonephritis with medullary necrosis |
| CKD | 57926 | K013.12 | Steroid sensitive nephrotic syndrome |
| CKD | 58750 | K01x300 | Nephrotic syndrome in polyarteritis nodosa |
| CKD | 60960 | K02y.00 | Other chronic glomerulonephritis |
| CKD | 61494 | K022.00 | Chronic membranoproliferative glomerulonephritis |

| | | | |
|---|---|---|---|
| CKD | 63615 | K02yz00 | Other chronic glomerulonephritis NOS |
| CKD | 63786 | K01w.00 | Congenital nephrotic syndrome |
| CKD | 65064 | K023.00 | Chronic rapidly progressive glomerulonephritis |
| CKD | 65400 | K02y300 | Chronic diffuse glomerulonephritis |
| CKD | 72303 | K01w000 | Finnish nephrosis syndrome |
| CKD | 94373 | K01y.00 | Nephrotic syndrome with other pathological kidney lesions |
| CKD | 94793 | 1Z1B.00 | Chronic kidney disease stage 3 with proteinuria |
| CKD | 94965 | 1Z15.00 | Chronic kidney disease stage 3A |
| CKD | 95122 | 1Z1H.00 | Chronic kidney disease stage 4 with proteinuria |
| CKD | 95123 | 1Z1C.00 | Chronic kidney disease stage 3 without proteinuria |
| CKD | 95175 | 1Z1E.00 | Chronic kidney disease stage 3A without proteinuria |
| CKD | 95177 | 1Z1G.00 | Chronic kidney disease stage 3B without proteinuria |
| CKD | 95178 | 1Z1F.00 | Chronic kidney disease stage 3B with proteinuria |
| CKD | 95179 | 1Z16.00 | Chronic kidney disease stage 3B |
| CKD | 95405 | 1Z1L.00 | Chronic kidney disease stage 5 without proteinuria |
| CKD | 95406 | 1Z1J.00 | Chronic kidney disease stage 4 without proteinuria |
| CKD | 95408 | 1Z1D.00 | Chronic kidney disease stage 3A with proteinuria |
| CKD | 95508 | 1Z1K.00 | Chronic kidney disease stage 5 with proteinuria |
| CKD | 97758 | K02y000 | Chronic glomerulonephritis + diseases EC |
| CKD | 99201 | K01x200 | Nephrotic syndrome in malaria |
| CKD | 99631 | K100000 | Chronic pyelonephritis without medullary necrosis |
| CKD | 99644 | K012.00 | Nephrotic syndrome+membranoproliferative glomerulonephritis |
| Dyslipidaemia | 339 | C320.00 | Pure hypercholesterolaemia |
| Dyslipidaemia | 637 | C324.00 | Hyperlipidaemia NOS |
| Dyslipidaemia | 1173 | C321.00 | Pure hyperglyceridaemia |
| Dyslipidaemia | 1391 | C327100 | Gaucher's disease |
| Dyslipidaemia | 1983 | C325z00 | Lipoprotein deficiency NOS |
| Dyslipidaemia | 3386 | C320000 | Familial hypercholesterolaemia |
| Dyslipidaemia | 3484 | C320.11 | Familial hypercholesterolaemia |
| Dyslipidaemia | 4833 | C322.13 | Xanthoma tuberosum |
| Dyslipidaemia | 5396 | C32y600 | Lipomatosis NEC |
| Dyslipidaemia | 5791 | C322.00 | Mixed hyperlipidaemia |
| Dyslipidaemia | 7447 | C320z00 | Pure hypercholesterolaemia NOS |
| Dyslipidaemia | 12439 | C321000 | Hypertriglyceridaemia |
| Dyslipidaemia | 13228 | C32..00 | Disorders of lipoid metabolism |
| Dyslipidaemia | 16290 | C325300 | A-beta-lipoproteinaemia |

| | | | |
|---|---|---|---|
| Dyslipidaemia | 16306 | C325.00 | Lipoprotein deficiencies |
| Dyslipidaemia | 16534 | C32yz00 | Other disorder of lipoid metabolism NOS |
| Dyslipidaemia | 18435 | C32y011 | Hoffa's Disease |
| Dyslipidaemia | 18472 | C32y500 | Steatosis |
| Dyslipidaemia | 18708 | C32..11 | Disorder of cholesterol metabolism |
| Dyslipidaemia | 19644 | C326.00 | Lipodystrophy |
| Dyslipidaemia | 20260 | C327z11 | Xanthomatosis, familial |
| Dyslipidaemia | 24161 | C327411 | Fabry's disease |
| Dyslipidaemia | 24660 | C327413 | Anderson-Fabry disease |
| Dyslipidaemia | 26019 | C320200 | Hyperlipidaemia, group A |
| Dyslipidaemia | 26037 | C32y511 | Hepatic familial steatosis |
| Dyslipidaemia | 28803 | C326000 | Progressive lipodystrophy |
| Dyslipidaemia | 30566 | C327.00 | Lipidoses |
| Dyslipidaemia | 33578 | C327311 | Primary familial xanthomatosis |
| Dyslipidaemia | 34146 | C325100 | Hypo-alpha-lipoproteinaemia |
| Dyslipidaemia | 34224 | C320300 | Low-density-lipoprotein-type (LDL) hyperlipoproteinaemia |
| Dyslipidaemia | 34825 | C320100 | Hyperbetalipoproteinaemia |
| Dyslipidaemia | 35063 | C323.00 | Hyperchylomicronaemia |
| Dyslipidaemia | 35717 | C327.12 | Fabry's disease |
| Dyslipidaemia | 37273 | C320400 | Fredrickson's hyperlipoproteinaemia, type IIa |
| Dyslipidaemia | 39699 | C325000 | High density lipoid deficiency |
| Dyslipidaemia | 39783 | C32z.00 | Disorder of lipoid metabolism NOS |
| Dyslipidaemia | 43714 | C32y000 | Liposynovitis prepatellaris |
| Dyslipidaemia | 46516 | C327200 | Niemann-Pick disease |
| Dyslipidaemia | 52992 | C322.11 | Fredrickson type IIb lipidaemia |
| Dyslipidaemia | 53091 | C320y00 | Other specified pure hypercholesterolaemia |
| Dyslipidaemia | 53527 | C32y300 | Pelvic lipomatosis |
| Dyslipidaemia | 54499 | C321.11 | Fredrickson type IV lipidaemia |
| Dyslipidaemia | 55432 | C32y400 | Lipase deficiency |
| Dyslipidaemia | 55855 | C320.12 | Fredrickson type IIa lipidaemia |
| Dyslipidaemia | 56796 | C326z00 | Lipodystrophy NOS |
| Dyslipidaemia | 59095 | C320.13 | Low density lipoproteinaemia |
| Dyslipidaemia | 59564 | C322.12 | Fredrickson type III lipidaemia |
| Dyslipidaemia | 64718 | C323.11 | Burger - Grute syndrome |
| Dyslipidaemia | 67948 | C32y200 | Lipoid dermatoarthritis |
| Dyslipidaemia | 68446 | C326.11 | Barraquer - Simons disease |

| | | | |
|---|---|---|---|
| Dyslipidaemia | 68741 | C32y.00 | Other disorders of lipoid metabolism |
| Dyslipidaemia | 69609 | C325311 | Bassen-Kornzweig disease |
| Dyslipidaemia | 69881 | C323.12 | Fredrickson type I lipaemia |
| Dyslipidaemia | 70793 | C325200 | Hypo-beta-lipoproteinaemia |
| Dyslipidaemia | 71157 | C327z00 | Lipidoses NOS |
| Dyslipidaemia | 71522 | C327400 | Alpha-galactosidase A deficiency |
| Dyslipidaemia | 72421 | C32y100 | Launois-Bensaude's lipomatosis |
| Dyslipidaemia | 91603 | C325.11 | Tangier disease |
| Dyslipidaemia | 93886 | C327412 | Anderson's disease |
| Dyslipidaemia | 95952 | C328.00 | Dyslipidaemia |
| Dyslipidaemia | 97989 | C320500 | Familial defective apolipoprotein B-100 |
| Dyslipidaemia | 99456 | C321.12 | Very low density lipoprotinaemia |
| Dyslipidaemia | 102390 | C322000 | Familial combined hyperlipidaemia |
| Dyslipidaemia | 102958 | C320600 | Polygenic hypercholesterolaemia |
| Dyslipidaemia | 104941 | C323.13 | Fredrickson type V lipaemia |
| Dyslipidaemia | 107252 | C329.00 | Hypercholesterolaemia |
| Dyslipidaemia | 109868 | C327300 | Wolman disease |
| Heart failure | 398 | G580.00 | Congestive heart failure |
| Heart failure | 884 | G581.00 | Left ventricular failure |
| Heart failure | 1223 | G58..11 | Cardiac failure |
| Heart failure | 2062 | G58..00 | Heart failure |
| Heart failure | 2906 | G580.11 | Congestive cardiac failure |
| Heart failure | 4024 | G58z.00 | Heart failure NOS |
| Heart failure | 5255 | G581000 | Acute left ventricular failure |
| Heart failure | 5942 | G581.13 | Impaired left ventricular function |
| Heart failure | 9524 | G580.14 | Biventricular failure |
| Heart failure | 9913 | 1O1..00 | Heart failure confirmed |
| Heart failure | 10079 | G580.12 | Right heart failure |
| Heart failure | 10154 | G580.13 | Right ventricular failure |
| Heart failure | 11424 | G580300 | Compensated cardiac failure |
| Heart failure | 17278 | G58z.12 | Cardiac failure NOS |
| Heart failure | 20822 | Q48y100 | Congenital cardiac failure |
| Heart failure | 21837 | G232.00 | Hypertensive heart&renal dis wth (congestive) heart failure |
| Heart failure | 22262 | G1yz100 | Rheumatic left ventricular failure |
| Heart failure | 23481 | G581.11 | Asthma - cardiac |
| Heart failure | 23566 | Q490.00 | Neonatal cardiac failure |

| | | | |
|---|---|---|---|
| Heart failure | 23707 | G580000 | Acute congestive heart failure |
| Heart failure | 27884 | G580200 | Decompensated cardiac failure |
| Heart failure | 27964 | G582.00 | Acute heart failure |
| Heart failure | 32671 | G580100 | Chronic congestive heart failure |
| Heart failure | 43618 | G581.12 | Pulmonary oedema - acute |
| Heart failure | 57987 | G234.00 | Hyperten heart&renal dis+both(congestv)heart and renal fail |
| Hypertension | 204 | G2...00 | Hypertensive disease |
| Hypertension | 351 | G20..11 | High blood pressure |
| Hypertension | 799 | G20..00 | Essential hypertension |
| Hypertension | 1894 | G201.00 | Benign essential hypertension |
| Hypertension | 3712 | G20z.11 | Hypertension NOS |
| Hypertension | 4372 | G202.00 | Systolic hypertension |
| Hypertension | 7057 | G2z..00 | Hypertensive disease NOS |
| Hypertension | 7329 | G24..00 | Secondary hypertension |
| Hypertension | 10818 | G20z.00 | Essential hypertension NOS |
| Hypertension | 15377 | G200.00 | Malignant essential hypertension |
| Hypertension | 16059 | G24z.00 | Secondary hypertension NOS |
| Hypertension | 18765 | G2y..00 | Other specified hypertensive disease |
| Hypertension | 25371 | G241000 | Secondary benign renovascular hypertension |
| Hypertension | 31341 | G24z100 | Hypertension secondary to drug |
| Hypertension | 31387 | G24z000 | Secondary renovascular hypertension NOS |
| Hypertension | 31755 | G240.00 | Secondary malignant hypertension |
| Hypertension | 34744 | G244.00 | Hypertension secondary to endocrine disorders |
| Hypertension | 42229 | G24zz00 | Secondary hypertension NOS |
| Hypertension | 51635 | G241z00 | Secondary benign hypertension NOS |
| Hypertension | 57288 | G241.00 | Secondary benign hypertension |
| Hypertension | 59383 | G240000 | Secondary malignant renovascular hypertension |
| Hypertension | 69753 | Gyu2.00 | [X]Hypertensive diseases |
| Hypertension | 73293 | G240z00 | Secondary malignant hypertension NOS |
| Hypertension | 83473 | G203.00 | Diastolic hypertension |
| Hypertension | 102458 | Gyu2000 | [X]Other secondary hypertension |
| MI | 241 | G30..00 | Acute myocardial infarction |
| MI | 1204 | G30..14 | Heart attack |
| MI | 1677 | G30..15 | MI - acute myocardial infarction |
| MI | 1678 | G308.00 | Inferior myocardial infarction NOS |
| MI | 2491 | G30..12 | Coronary thrombosis |

| | | | |
|---|---|---|---|
| MI | 3704 | G307.00 | Acute subendocardial infarction |
| MI | 5387 | G301.00 | Other specified anterior myocardial infarction |
| MI | 7783 | 323..00 | ECG: myocardial infarction |
| MI | 8935 | G302.00 | Acute inferolateral infarction |
| MI | 9507 | G307000 | Acute non-Q wave infarction |
| MI | 10562 | G307100 | Acute non-ST segment elevation myocardial infarction |
| MI | 12139 | G300.00 | Acute anterolateral infarction |
| MI | 12229 | G30X000 | Acute ST segment elevation myocardial infarction |
| MI | 13566 | G30..11 | Attack - heart |
| MI | 13571 | G30..16 | Thrombosis - coronary |
| MI | 14658 | G30z.00 | Acute myocardial infarction NOS |
| MI | 14897 | G301z00 | Anterior myocardial infarction NOS |
| MI | 14898 | G305.00 | Lateral myocardial infarction NOS |
| MI | 16408 | G32..11 | Healed myocardial infarction |
| MI | 17133 | G30A.00 | Mural thrombosis |
| MI | 17464 | G32..12 | Personal history of myocardial infarction |
| MI | 17689 | G30..17 | Silent myocardial infarction |
| MI | 17872 | G301100 | Acute anteroseptal infarction |
| MI | 18842 | G35..00 | Subsequent myocardial infarction |
| MI | 23579 | G310.00 | Postmyocardial infarction syndrome |
| MI | 23708 | G361.00 | Atrial septal defect/curr comp folow acut myocardal infarct |
| MI | 23892 | G304.00 | Posterior myocardial infarction NOS |
| MI | 24126 | G360.00 | Haemopericardium/current comp folow acut myocard infarct |
| MI | 28736 | G30y000 | Acute atrial infarction |
| MI | 29643 | G303.00 | Acute inferoposterior infarction |
| MI | 29758 | G30X.00 | Acute transmural myocardial infarction of unspecif site |
| MI | 30330 | G309.00 | Acute Q-wave infarct |
| MI | 30421 | G30..13 | Cardiac rupture following myocardial infarction (MI) |
| MI | 32272 | G38..00 | Postoperative myocardial infarction |
| MI | 32854 | G30B.00 | Acute posterolateral myocardial infarction |
| MI | 34803 | G30y.00 | Other acute myocardial infarction |
| MI | 36423 | G36..00 | Certain current complication follow acute myocardial infarct |
| MI | 37657 | G362.00 | Ventric septal defect/curr comp fol acut myocardal infarctn |
| MI | 38609 | G351.00 | Subsequent myocardial infarction of inferior wall |
| MI | 40429 | G301000 | Acute anteroapical infarction |
| MI | 41221 | G30y200 | Acute septal infarction |

| | | | |
|---|---|---|---|
| MI | 41835 | G384.00 | Postoperative subendocardial myocardial infarction |
| MI | 45809 | G350.00 | Subsequent myocardial infarction of anterior wall |
| MI | 46017 | G30yz00 | Other acute myocardial infarction NOS |
| MI | 46112 | G380.00 | Postoperative transmural myocardial infarction anterior wall |
| MI | 46166 | G35X.00 | Subsequent myocardial infarction of unspecified site |
| MI | 46276 | G381.00 | Postoperative transmural myocardial infarction inferior wall |
| MI | 59032 | 323Z.00 | ECG: myocardial infarct NOS |
| MI | 59940 | G364.00 | Ruptur chordae tendinae/curr comp fol acute myocard infarct |
| MI | 61670 | 889A.00 | Diab mellit insulin-glucose infus acute myocardial infarct |
| MI | 62626 | G30y100 | Acute papillary muscle infarction |
| MI | 63467 | G306.00 | True posterior myocardial infarction |
| MI | 68748 | G38z.00 | Postoperative myocardial infarction, unspecified |
| MI | 69474 | G365.00 | Rupture papillary muscle/curr comp fol acute myocard infarct |
| MI | 72562 | G353.00 | Subsequent myocardial infarction of other sites |
| MI | 96838 | Gyu3400 | [X]Acute transmural myocardial infarction of unspecif site |
| MI | 99991 | Gyu3600 | [X]Subsequent myocardial infarction of unspecified site |
| MI | 106812 | G383.00 | Postoperative transmural myocardial infarction unspec site |
| MI | 109035 | Gyu3500 | [X]Subsequent myocardial infarction of other sites |
| Osteoarthritis | 396 | N05..11 | Osteoarthritis |
| Osteoarthritis | 639 | N05z211 | Elbow osteoarthritis NOS |
| Osteoarthritis | 658 | N05z400 | Osteoarthritis NOS, of the hand |
| Osteoarthritis | 665 | N05z611 | Knee osteoarthritis NOS |
| Osteoarthritis | 1104 | N053512 | Hip osteoarthitis NOS |
| Osteoarthritis | 1296 | N053611 | Patellofemoral osteoarthritis |
| Osteoarthritis | 1312 | N05z712 | Foot osteoarthritis NOS |
| Osteoarthritis | 1509 | N05z.11 | Joint degeneration |
| Osteoarthritis | 1959 | N05z412 | Thumb osteoarthritis NOS |
| Osteoarthritis | 2209 | N05z511 | Hip osteoarthritis NOS |
| Osteoarthritis | 2229 | N05zB00 | Osteoarthritis NOS, of acromioclavicular joint |
| Osteoarthritis | 2487 | N05zL00 | Osteoarthritis NOS, of knee |
| Osteoarthritis | 3057 | N05..00 | Osteoarthritis and allied disorders |
| Osteoarthritis | 3147 | N05z100 | Osteoarthritis NOS, of shoulder region |
| Osteoarthritis | 3814 | N05zA00 | Osteoarthritis NOS, of sternoclavicular joint |
| Osteoarthritis | 4015 | N050111 | Heberdens' nodes |
| Osteoarthritis | 4353 | N050.00 | Generalised osteoarthritis - OA |
| Osteoarthritis | 4461 | N053700 | Localised osteoarthritis, unspecified, of the ankle and foot |

| | | | |
|---|---|---|---|
| Osteoarthritis | 4490 | N05z411 | Finger osteoarthritis NOS |
| Osteoarthritis | 4878 | N05z713 | Toe osteoarthritis NOS |
| Osteoarthritis | 4967 | N05z500 | Osteoarthritis NOS, pelvic region/thigh |
| Osteoarthritis | 5776 | N05z.00 | Osteoarthritis NOS |
| Osteoarthritis | 5802 | N05z900 | Osteoarthritis NOS, of shoulder |
| Osteoarthritis | 6812 | N05zJ00 | Osteoarthritis NOS, of hip |
| Osteoarthritis | 6887 | N05zS00 | Osteoarthritis NOS, of 1st MTP joint |
| Osteoarthritis | 7866 | N05zF00 | Osteoarthritis NOS, of MCP joint |
| Osteoarthritis | 8202 | N05zN00 | Osteoarthritis NOS, of ankle |
| Osteoarthritis | 9010 | N05zT00 | Osteoarthritis NOS, of lesser MTP joint |
| Osteoarthritis | 9649 | N05zE00 | Osteoarthritis NOS, of wrist |
| Osteoarthritis | 9681 | N05zH00 | Osteoarthritis NOS, of DIP joint of finger |
| Osteoarthritis | 11032 | N05zG00 | Osteoarthritis NOS, of PIP joint of finger |
| Osteoarthritis | 11256 | N050500 | Secondary multiple arthrosis |
| Osteoarthritis | 15052 | N05z800 | Osteoarthritis NOS, other specified site |
| Osteoarthritis | 15144 | N05z600 | Osteoarthritis NOS, of the lower leg |
| Osteoarthritis | 15206 | N05z311 | Wrist osteoarthritis NOS |
| Osteoarthritis | 15441 | N053100 | Localised osteoarthritis, unspecified, of shoulder region |
| Osteoarthritis | 15447 | N05z700 | Osteoarthritis NOS, of ankle and foot |
| Osteoarthritis | 15839 | N051500 | Localised, primary osteoarthritis of the pelvic region/thigh |
| Osteoarthritis | 16242 | N053400 | Localised osteoarthritis, unspecified, of the hand |
| Osteoarthritis | 18112 | N053800 | Localised osteoarthritis, unspecified, of other spec site |
| Osteoarthritis | 18602 | N051F00 | Localised, primary osteoarthritis of elbow |
| Osteoarthritis | 19713 | N05zC00 | Osteoarthritis NOS, of elbow |
| Osteoarthritis | 20472 | N051800 | Localised, primary osteoarthritis of other specified site |
| Osteoarthritis | 20626 | N053500 | Localised osteoarthritis, unspecified, pelvic region/thigh |
| Osteoarthritis | 20660 | N051z00 | Localised, primary osteoarthritis NOS |
| Osteoarthritis | 21159 | N051600 | Localised, primary osteoarthritis of the lower leg |
| Osteoarthritis | 21177 | N053900 | Arthrosis of first carpometacarpal joint, unspecified |
| Osteoarthritis | 21350 | N051400 | Localised, primary osteoarthritis of the hand |
| Osteoarthritis | 21528 | N054.00 | Oligoarticular osteoarthritis, unspecified |
| Osteoarthritis | 23638 | N052400 | Localised, secondary osteoarthritis of the hand |
| Osteoarthritis | 23646 | N050400 | Primary generalized osteoarthrosis |
| Osteoarthritis | 23676 | N050200 | Generalised osteoarthritis of multiple sites |
| Osteoarthritis | 24022 | N051100 | Localised, primary osteoarthritis of the shoulder region |
| Osteoarthritis | 24146 | N051B00 | Primary gonarthrosis, bilateral |

| | | | |
|---|---|---|---|
| Osteoarthritis | 24152 | N05z300 | Osteoarthritis NOS, of the forearm |
| Osteoarthritis | 24217 | N051200 | Localised, primary osteoarthritis of the upper arm |
| Osteoarthritis | 24287 | N051900 | Primary coxarthrosis, bilateral |
| Osteoarthritis | 24392 | N052A00 | Post-traumatic gonarthrosis, bilateral |
| Osteoarthritis | 24432 | N050700 | Heberden's nodes with arthropathy |
| Osteoarthritis | 24958 | N051D00 | Localised, primary osteoarthritis of the wrist |
| Osteoarthritis | 25793 | N051700 | Localised, primary osteoarthritis of the ankle and foot |
| Osteoarthritis | 25812 | N051A00 | Coxarthrosis resulting from dysplasia, bilateral |
| Osteoarthritis | 27834 | N05zU00 | Osteoarthritis NOS, of IP joint of toe |
| Osteoarthritis | 27972 | N05zz00 | Osteoarthritis NOS |
| Osteoarthritis | 28908 | N051E00 | Localised, primary osteoarthritis of toe |
| Osteoarthritis | 31200 | N053z00 | Localised osteoarthritis, unspecified, NOS |
| Osteoarthritis | 32839 | N051.00 | Localised, primary osteoarthritis |
| Osteoarthritis | 32891 | N052800 | Localised, secondary osteoarthritis of other specified site |
| Osteoarthritis | 33479 | N052600 | Localised, secondary osteoarthritis of the lower leg |
| Osteoarthritis | 33574 | N052100 | Localised, secondary osteoarthritis of the shoulder region |
| Osteoarthritis | 34023 | N05zK00 | Osteoarthritis NOS, of sacro-iliac joint |
| Osteoarthritis | 34035 | N052700 | Localised, secondary osteoarthritis of the ankle and foot |
| Osteoarthritis | 34122 | N053.00 | Localised osteoarthritis, unspecified |
| Osteoarthritis | 34804 | N053600 | Localised osteoarthritis, unspecified, of the lower leg |
| Osteoarthritis | 34806 | N051300 | Localised, primary osteoarthritis of the forearm |
| Osteoarthritis | 34867 | N050z00 | Generalised osteoarthritis NOS |
| Osteoarthritis | 35527 | N05z000 | Osteoarthritis NOS, of unspecified site |
| Osteoarthritis | 35919 | N050112 | Bouchards' nodes |
| Osteoarthritis | 36182 | N051C00 | Primary arthrosis of first carpometacarpal joints, bilateral |
| Osteoarthritis | 36327 | N050100 | Generalised osteoarthritis of the hand |
| Osteoarthritis | 38018 | N050300 | Bouchard's nodes with arthropathy |
| Osteoarthritis | 38019 | N050600 | Erosive osteoarthrosis |
| Osteoarthritis | 38631 | N050000 | Generalised osteoarthritis of unspecified site |
| Osteoarthritis | 40972 | N05zP00 | Osteoarthritis NOS, of subtalar joint |
| Osteoarthritis | 41088 | N052200 | Localised, secondary osteoarthritis of the upper arm |
| Osteoarthritis | 41090 | N054600 | Oligoarticular osteoarthritis, unspecified, of lower leg |
| Osteoarthritis | 41985 | N054800 | Oligoarticular osteoarthritis, unspecified, other spec sites |
| Osteoarthritis | 42045 | N052.00 | Localised, secondary osteoarthritis |
| Osteoarthritis | 44041 | N052500 | Localised, secondary osteoarthritis of pelvic region/thigh |
| Osteoarthritis | 45815 | N052300 | Localised, secondary osteoarthritis of the forearm |

| | | | |
|---|---|---|---|
| Osteoarthritis | 48214 | N054000 | Oligoarticular osteoarthritis, unspec, of unspecified sites |
| Osteoarthritis | 49545 | N053000 | Localised osteoarthritis, unspecified, of unspecified site |
| Osteoarthritis | 50470 | N052C00 | Post-traumatic gonarthrosis, unilateral |
| Osteoarthritis | 50848 | N05z200 | Osteoarthritis NOS, of the upper arm |
| Osteoarthritis | 52095 | N054100 | Oligoarticular osteoarthritis, unspecified, of shoulder |
| Osteoarthritis | 52897 | N05z711 | Ankle osteoarthritis NOS |
| Osteoarthritis | 52925 | N053511 | Otto's pelvis |
| Osteoarthritis | 53858 | N054z00 | Osteoarthritis of more than one site, unspecified, NOS |
| Osteoarthritis | 54224 | N051000 | Localised, primary osteoarthritis of unspecified site |
| Osteoarthritis | 54350 | N05zR00 | Osteoarthritis NOS, of other tarsal joint |
| Osteoarthritis | 55388 | N05zQ00 | Osteoarthritis NOS, of talonavicular joint |
| Osteoarthritis | 57267 | N054900 | Oligoarticular osteoarthritis, unspecified, multiple sites |
| Osteoarthritis | 57912 | N052z00 | Localised, secondary osteoarthritis NOS |
| Osteoarthritis | 59616 | N054400 | Oligoarticular osteoarthritis, unspecified, of hand |
| Osteoarthritis | 59637 | N053200 | Localised osteoarthritis, unspecified, of the upper arm |
| Osteoarthritis | 60183 | N052B00 | Post-traumatic arthrosis of first carpometacarpal jt bilat |
| Osteoarthritis | 60537 | N053300 | Localised osteoarthritis, unspecified, of the forearm |
| Osteoarthritis | 64503 | N052900 | Post-traumatic coxarthrosis, bilateral |
| Osteoarthritis | 65748 | N05zD00 | Osteoarthritis NOS, of distal radio-ulnar joint |
| Osteoarthritis | 68648 | N054500 | Oligoarticular osteoarthritis, unspecified, of pelvis/thigh |
| Osteoarthritis | 68712 | N052000 | Localised, secondary osteoarthritis of unspecified site |
| Osteoarthritis | 70425 | N05zM00 | Osteoarthritis NOS, of tibio-fibular joint |
| Osteoarthritis | 72109 | N054700 | Oligoarticular osteoarthritis, unspecified, of ankle/foot |
| Osteoarthritis | 97073 | N054200 | Oligoarticular osteoarthritis, unspecified, of upper arm |
| Osteoarthritis | 101479 | N052511 | Coxae malum senilis |
| Osteoarthritis | 106678 | N051G00 | Osteoarthritis of spinal facet joint |
| Sleep apnea | 2506 | R005311 | [D]Sleep apnea syndrome |
| Sleep apnea | 7603 | Fy03.00 | Sleep apnea |
| Sleep apnea | 8148 | Fy03.11 | Obstructive sleep apnea |
| Sleep apnea | 20438 | R005312 | [D]Syndrome sleep apnea |
| Sleep apnea | 20748 | H5B0.00 | Obstructive sleep apnea |
| Sleep apnea | 23779 | H5B..00 | Sleep apnea |
| Sleep apnea | 26871 | Q318.00 | Primary sleep apnea of newborn |
| Sleep apnea | 36301 | R005300 | [D]Hypersomnia with sleep apnea |
| Sleep apnea | 48539 | R005100 | [D]Insomnia with sleep apnea |
| Stroke | 1298 | G66..11 | CVA unspecified |

| | | | |
|---|---|---|---|
| Stroke | 1469 | G66..00 | Stroke and cerebrovascular accident unspecified |
| Stroke | 6116 | G66..13 | CVA - Cerebrovascular accident unspecified |
| Stroke | 6155 | G64..13 | Stroke due to cerebral arterial occlusion |
| Stroke | 6228 | G68X.00 | Sequelae of stroke,not specfd as h'morrhage or infarction |
| Stroke | 6253 | G66..12 | Stroke unspecified |
| Stroke | 7780 | G667.00 | Left sided CVA |
| Stroke | 8443 | G663.00 | Brain stem stroke syndrome |
| Stroke | 12833 | G668.00 | Right sided CVA |
| Stroke | 13707 | 8HBJ.00 | Stroke / transient ischaemic attack referral |
| Stroke | 16956 | G669.00 | Cerebral palsy, not congenital or infantile, acute |
| Stroke | 17322 | G664.00 | Cerebellar stroke syndrome |
| Stroke | 18604 | G61..12 | Stroke due to intracerebral haemorrhage |
| Stroke | 18689 | G660.00 | Middle cerebral artery syndrome |
| Stroke | 18804 | 8HTQ.00 | Referral to stroke clinic |
| Stroke | 19260 | G662.00 | Posterior cerebral artery syndrome |
| Stroke | 19280 | G661.00 | Anterior cerebral artery syndrome |
| Stroke | 32959 | 9N0p.00 | Seen in stroke clinic |
| Stroke | 33499 | G665.00 | Pure motor lacunar syndrome |
| Stroke | 42248 | ZLEP.00 | Discharge from stroke serv |
| Stroke | 51767 | G666.00 | Pure sensory lacunar syndrome |
| Stroke | 52246 | 13YA.00 | Stroke group member |
| Stroke | 55351 | 7P24200 | Delivery of rehabilitation for stroke |
| Stroke | 56279 | L440.12 | Stroke in the puerperium |
| Stroke | 56458 | 8HHM.00 | Ref to multidisciplinary stroke function improvement service |
| Stroke | 100639 | 1M4..00 | Central post-stroke pain |
| Stroke | 104505 | 662M200 | Stroke initial post discharge review |
| Stroke | 104638 | 8IEC.00 | Ref multidisciplinary stroke function improvement declined |
| Stroke | 105520 | 8Hd6.00 | Admission to stroke unit |
| Stroke | 110337 | Gyu6C00 | [X]Sequelae of stroke,not specfd as h'morrhage or infarction |
| T2D | 506 | C100112 | Non-insulin dependent diabetes mellitus |
| T2D | 758 | C10F.00 | Type 2 diabetes mellitus |
| T2D | 1407 | C10FJ00 | Insulin treated Type 2 diabetes mellitus |
| T2D | 1684 | 66A4.00 | Diabetic on oral treatment |
| T2D | 4513 | C109.00 | Non-insulin dependent diabetes mellitus |
| T2D | 5884 | C109.11 | NIDDM - Non-insulin dependent diabetes mellitus |
| T2D | 7563 | 66A3.00 | Diabetic on diet only |

| | | | |
|---|---|---|---|
| T2D | 8403 | C109700 | Non-insulin dependent diabetes mellitus - poor control |
| T2D | 12640 | C10FC00 | Type 2 diabetes mellitus with nephropathy |
| T2D | 12736 | C10F500 | Type 2 diabetes mellitus with gangrene |
| T2D | 14803 | C100100 | Diabetes mellitus, adult onset, no mention of complication |
| T2D | 14889 | C100111 | Maturity onset diabetes |
| T2D | 17262 | C109600 | Non-insulin-dependent diabetes mellitus with retinopathy |
| T2D | 17859 | C109.12 | Type 2 diabetes mellitus |
| T2D | 18143 | C109G11 | Type II diabetes mellitus with arthropathy |
| T2D | 18209 | C109012 | Type 2 diabetes mellitus with renal complications |
| T2D | 18219 | C109.13 | Type II diabetes mellitus |
| T2D | 18264 | C109J12 | Insulin treated Type II diabetes mellitus |
| T2D | 18278 | C109J00 | Insulin treated Type 2 diabetes mellitus |
| T2D | 18390 | C10FM00 | Type 2 diabetes mellitus with persistent microalbuminuria |
| T2D | 18425 | C10FB00 | Type 2 diabetes mellitus with polyneuropathy |
| T2D | 18496 | C10F600 | Type 2 diabetes mellitus with retinopathy |
| T2D | 18777 | C10F000 | Type 2 diabetes mellitus with renal complications |
| T2D | 22884 | C10F.11 | Type II diabetes mellitus |
| T2D | 24458 | C109711 | Type II diabetes mellitus - poor control |
| T2D | 24693 | C109G00 | Non-insulin dependent diabetes mellitus with arthropathy |
| T2D | 24836 | C109C12 | Type 2 diabetes mellitus with nephropathy |
| T2D | 25591 | C10FQ00 | Type 2 diabetes mellitus with exudative maculopathy |
| T2D | 25627 | C10F700 | Type 2 diabetes mellitus - poor control |
| T2D | 26054 | C10FL00 | Type 2 diabetes mellitus with persistent proteinuria |
| T2D | 28769 | 66AV.00 | Diabetic on insulin and oral treatment |
| T2D | 29979 | C109900 | Non-insulin-dependent diabetes mellitus without complication |
| T2D | 31310 | C108900 | Insulin dependent diabetes maturity onset |
| T2D | 32627 | C10FN00 | Type 2 diabetes mellitus with ketoacidosis |
| T2D | 33807 | C107200 | Diabetes mellitus, adult with gangrene |
| T2D | 34268 | C10F200 | Type 2 diabetes mellitus with neurological complications |
| T2D | 34450 | C10FK00 | Hyperosmolar non-ketotic state in type 2 diabetes mellitus |
| T2D | 34912 | C109400 | Non-insulin dependent diabetes mellitus with ulcer |
| T2D | 35105 | C104100 | Diabetes mellitus, adult onset, with renal manifestation |
| T2D | 35385 | C10FH00 | Type 2 diabetes mellitus with neuropathic arthropathy |
| T2D | 36633 | C109K00 | Hyperosmolar non-ketotic state in type 2 diabetes mellitus |
| T2D | 37648 | C109J11 | Insulin treated non-insulin dependent diabetes mellitus |
| T2D | 37806 | C10FF00 | Type 2 diabetes mellitus with peripheral angiopathy |

| | | | |
|---|---|---|---|
| T2D | 39317 | C106100 | Diabetes mellitus, adult onset, + neurological manifestation |
| T2D | 40401 | C109500 | Non-insulin dependent diabetes mellitus with gangrene |
| T2D | 41389 | C105100 | Diabetes mellitus, adult onset, + ophthalmic manifestation |
| T2D | 42762 | C109612 | Type 2 diabetes mellitus with retinopathy |
| T2D | 43139 | C102100 | Diabetes mellitus, adult onset, with hyperosmolar coma |
| T2D | 43227 | C10F311 | Type II diabetes mellitus with multiple complications |
| T2D | 43785 | C109D00 | Non-insulin dependent diabetes mellitus with hypoglyca coma |
| T2D | 44779 | C109E12 | Type 2 diabetes mellitus with diabetic cataract |
| T2D | 44982 | C10FE00 | Type 2 diabetes mellitus with diabetic cataract |
| T2D | 45467 | C109B00 | Non-insulin dependent diabetes mellitus with polyneuropathy |
| T2D | 45913 | C109712 | Type 2 diabetes mellitus - poor control |
| T2D | 45919 | C109212 | Type 2 diabetes mellitus with neurological complications |
| T2D | 46917 | C10FD00 | Type 2 diabetes mellitus with hypoglycaemic coma |
| T2D | 47315 | C10F711 | Type II diabetes mellitus - poor control |
| T2D | 47321 | C10F100 | Type 2 diabetes mellitus with ophthalmic complications |
| T2D | 47409 | C109B11 | Type II diabetes mellitus with polyneuropathy |
| T2D | 47816 | C109H11 | Type II diabetes mellitus with neuropathic arthropathy |
| T2D | 47954 | C10F900 | Type 2 diabetes mellitus without complication |
| T2D | 48192 | C109E11 | Type II diabetes mellitus with diabetic cataract |
| T2D | 49074 | C10F400 | Type 2 diabetes mellitus with ulcer |
| T2D | 49655 | C10F611 | Type II diabetes mellitus with retinopathy |
| T2D | 49869 | C109G12 | Type 2 diabetes mellitus with arthropathy |
| T2D | 50225 | C109011 | Type II diabetes mellitus with renal complications |
| T2D | 50429 | C109100 | Non-insulin-dependent diabetes mellitus with ophthalm comps |
| T2D | 50527 | C10FB11 | Type II diabetes mellitus with polyneuropathy |
| T2D | 50813 | C109A11 | Type II diabetes mellitus with mononeuropathy |
| T2D | 51756 | C10FP00 | Type 2 diabetes mellitus with ketoacidotic coma |
| T2D | 52303 | C109000 | Non-insulin-dependent diabetes mellitus with renal comps |
| T2D | 53392 | C10F911 | Type II diabetes mellitus without complication |
| T2D | 54856 | C101100 | Diabetes mellitus, adult onset, with ketoacidosis |
| T2D | 54899 | C109F11 | Type II diabetes mellitus with peripheral angiopathy |
| T2D | 55075 | C109411 | Type II diabetes mellitus with ulcer |
| T2D | 55842 | C109200 | Non-insulin-dependent diabetes mellitus with neuro comps |
| T2D | 56268 | C109D11 | Type II diabetes mellitus with hypoglycaemic coma |
| T2D | 57278 | C10F011 | Type II diabetes mellitus with renal complications |
| T2D | 58604 | C109611 | Type II diabetes mellitus with retinopathy |

| | | | |
|---|---|---|---|
| T2D | 59253 | C10FG00 | Type 2 diabetes mellitus with arthropathy |
| T2D | 59365 | C109C00 | Non-insulin dependent diabetes mellitus with nephropathy |
| T2D | 59725 | C109111 | Type II diabetes mellitus with ophthalmic complications |
| T2D | 60699 | C109F12 | Type 2 diabetes mellitus with peripheral angiopathy |
| T2D | 60796 | C10FL11 | Type II diabetes mellitus with persistent proteinuria |
| T2D | 61071 | C109D12 | Type 2 diabetes mellitus with hypoglycaemic coma |
| T2D | 62107 | C109511 | Type II diabetes mellitus with gangrene |
| T2D | 62146 | C109300 | Non-insulin-dependent diabetes mellitus with multiple comps |
| T2D | 62674 | C10FA00 | Type 2 diabetes mellitus with mononeuropathy |
| T2D | 63357 | C107100 | Diabetes mellitus, adult, + peripheral circulatory disorder |
| T2D | 63371 | C10y100 | Diabetes mellitus, adult, + other specified manifestation |
| T2D | 63690 | C10FR00 | Type 2 diabetes mellitus with gastroparesis |
| T2D | 63762 | C10z100 | Diabetes mellitus, adult onset, + unspecified complication |
| T2D | 64571 | C109C11 | Type II diabetes mellitus with nephropathy |
| T2D | 64668 | C10FJ11 | Insulin treated Type II diabetes mellitus |
| T2D | 65267 | C10F300 | Type 2 diabetes mellitus with multiple complications |
| T2D | 65704 | C109412 | Type 2 diabetes mellitus with ulcer |
| T2D | 66965 | C109H12 | Type 2 diabetes mellitus with neuropathic arthropathy |
| T2D | 67905 | C109211 | Type II diabetes mellitus with neurological complications |
| T2D | 68843 | C103100 | Diabetes mellitus, adult onset, with ketoacidotic coma |
| T2D | 69278 | C109E00 | Non-insulin depend diabetes mellitus with diabetic cataract |
| T2D | 70316 | C109112 | Type 2 diabetes mellitus with ophthalmic complications |
| T2D | 72320 | C109A00 | Non-insulin dependent diabetes mellitus with mononeuropathy |
| T2D | 102201 | C10FC11 | Type II diabetes mellitus with nephropathy |
| T2D | 104323 | C10F511 | Type II diabetes mellitus with gangrene |
| T2D | 104639 | C10FF11 | Type II diabetes mellitus with peripheral angiopathy |
| T2D | 109103 | C109911 | Type II diabetes mellitus without complication |
| TIA | 504 | G65..00 | Transient cerebral ischaemia |
| TIA | 1433 | G65..12 | Transient ischaemic attack |
| TIA | 1895 | G65z.00 | Transient cerebral ischaemia NOS |
| TIA | 2417 | G65..13 | Vertebro-basilar insufficiency |
| TIA | 3132 | G65..11 | Drop attack |
| TIA | 5268 | G650.11 | Insufficiency - basilar artery |
| TIA | 6489 | G655.00 | Transient global amnesia |
| TIA | 10794 | G656.00 | Vertebrobasilar insufficiency |
| TIA | 13707 | 8HBJ.00 | Stroke / transient ischaemic attack referral |

| | | | |
|---|---|---|---|
| TIA | 15788 | G65zz00 | Transient cerebral ischaemia NOS |
| TIA | 16507 | G65z100 | Intermittent cerebral ischaemia |
| TIA | 19354 | G65y.00 | Other transient cerebral ischaemia |
| TIA | 21118 | G651000 | Vertebro-basilar artery syndrome |
| TIA | 23465 | G652.00 | Subclavian steal syndrome |
| TIA | 23942 | G650.00 | Basilar artery syndrome |
| TIA | 33377 | G651.00 | Vertebral artery syndrome |
| TIA | 44765 | G653.00 | Carotid artery syndrome hemispheric |
| TIA | 50594 | G654.00 | Multiple and bilateral precerebral artery syndromes |
| TIA | 55247 | G65z000 | Impending cerebral ischaemia |
| TIA | 100015 | 8CRB.00 | Transient ischaemic attack clinical management plan |
| TIA | 101251 | ZV12D00 | [V]Personal history of transient ischaemic attack |
| TIA | 105738 | G657.00 | Carotid territory transient ischaemic attack |
| Unstable angina | 1431 | G311.13 | Unstable angina |
| Unstable angina | 4656 | G311.11 | Crescendo angina |
| Unstable angina | 7347 | G311100 | Unstable angina |
| Unstable angina | 11983 | G311500 | Acute coronary syndrome |
| Unstable angina | 17307 | G311200 | Angina at rest |
| Unstable angina | 18118 | G311400 | Worsening angina |
| Unstable angina | 19655 | G311.14 | Angina at rest |
| Unstable angina | 34328 | G311300 | Refractory angina |
| Unstable angina | 36523 | G311.00 | Preinfarction syndrome |
| Unstable angina | 39655 | G311.12 | Impending infarction |
| Unstable angina | 54251 | G311z00 | Preinfarction syndrome NOS |
| Unstable angina | 55137 | G311011 | MI - myocardial infarction aborted |
| Unstable angina | 61072 | G311000 | Myocardial infarction aborted |

**Table S2 ICD-10 codes used to identify conditions and events in HES and ONS data**

| type | icd |
|---|---|
| Atrial fibrillation | I48.0 |
| Atrial fibrillation | I48.1 |
| Atrial fibrillation | I48.2 |
| CKD | N18.1 |
| CKD | N18.2 |
| CKD | N18.3 |
| CKD | N18.4 |
| CKD | N18.5 |
| CKD | N18.9 |
| Heart failure | I50.0 |
| Heart failure | I50.1 |
| Heart failure | I50.9 |
| MI | I21.0 |
| MI | I21.1 |
| MI | I21.2 |
| MI | I21.3 |
| MI | I21.4 |
| MI | I21.9 |
| MI | I22.0 |
| MI | I22.1 |
| MI | I22.8 |
| MI | I22.9 |
| Sleep apnea | G47.3 |
| Stroke | I60 |
| Stroke | I60.0 |
| Stroke | I60.2 |
| Stroke | I60.3 |
| Stroke | I60.4 |
| Stroke | I60.5 |
| Stroke | I60.6 |
| Stroke | I60.7 |
| Stroke | I60.8 |
| Stroke | I60.9 |
| Stroke | I61.0 |

| | |
|---|---|
| Stroke | I61.1 |
| Stroke | I61.2 |
| Stroke | I61.3 |
| Stroke | I61.4 |
| Stroke | I61.5 |
| Stroke | I61.6 |
| Stroke | I61.8 |
| Stroke | I61.9 |
| Stroke | I62.0 |
| Stroke | I62.1 |
| Stroke | I62.3 |
| Stroke | I62.9 |
| Stroke | I63.0 |
| Stroke | I63.1 |
| Stroke | I63.2 |
| Stroke | I63.4 |
| Stroke | I63.5 |
| Stroke | I63.6 |
| Stroke | I63.8 |
| Stroke | I63.9 |
| Stroke | I64 |
| TIA | G45.0 |
| TIA | G45.1 |
| TIA | G45.2 |
| TIA | G45.3 |
| TIA | G45.4 |
| TIA | G45.8 |
| TIA | G45.9 |
| Unstable angina | I20.0 |

**Table S3** Risks for each condition and event during the follow-up period.

| **Condition/event** | **BMI group (kg/m$^2$)** | | | | | **Sex (HRs for men, relative to women)** | **Smoking (HRs for ever, relative to never)** |
|---|---|---|---|---|---|---|---|
| | **Normal** | **25–29.9 Overweight** | **30–34.9 Obesity I** | **35–39.9 Obesity II** | **40–45 Obesity III** | | |
| **Type 2 diabetes** | 1.0 (ref) | 2.41 (2.37–2.45) | 5.17 (5.09–5.25) | 8.79 (8.63–8.95) | 12.4 (12.1–12.7) | 1.58 (1.56–1.59) | 1.25 (1.24–1.27) |
| **Asthma** | 1.0 (ref) | 1.28 (1.26–1.31) | 1.53 (1.50–1.57) | 1.73 (1.68–1.78) | 1.85 (1.77–1.93) | 0.72 (0.71–0.73) | 1.27 (1.25–1.29) |
| **Sleep apnea** | 1.0 (ref) | 2.22 (2.13–2.32) | 5.11 (4.90–5.33) | 10.5 (10.1–11.0) | 19.8 (18.9–20.8) | 3.22 (3.14–3.30) | 1.29 (1.27–1.33) |
| **Osteoarthritis** | 1.0 (ref) | 1.35 (1.33–1.36) | 1.68 (1.66–1.70) | 1.99 (1.96–2.02) | 2.31 (2.26–2.37) | 0.69 (0.68–0.69) | 1.04 (1.03–1.04) |
| **Heart failure** | 1.0 (ref) | 1.12 (1.11–1.14) | 1.57 (1.55–1.60) | 2.33 (2.28–2.39) | 3.46 (3.35–3.57) | 1.51 (1.50–1.53) | 1.40 (1.38–1.41) |
| **Chronic kidney disease** | 1.0 (ref) | 1.27 (1.26–1.28) | 1.59 (1.57–1.61) | 1.88 (1.86–1.91) | 2.29 (2.24–2.35) | 0.91 (0.91–0.92) | 1.14 (1.13–1.15) |
| **Hypertension** | 1.0 (ref) | 1.44 (1.43–1.45) | 1.99 (1.97–2.01) | 2.61 (2.58–2.64) | 3.21 (3.15–3.26) | 1.71 (1.70–1.72) | 1.13 (1.12–1.14) |
| **Dyslipidaemia** | 1.0 (ref) | 1.42 (1.41–1.43) | 1.83 (1.81–1.84) | 2.15 (2.13–2.18) | 2.45 (2.41–2.49) | 1.47 (1.46–1.48) | 1.28 (1.28–1.29) |
| **Atrial fibrillation** | 1.0 (ref) | 1.15 (1.14–1.17) | 1.51 (1.48–1.53) | 2.02 (1.97–2.06) | 2.78 (2.68–2.87) | 1.55 (1.53–1.57) | 1.08 (1.06–1.09) |
| **Unstable angina/MI** | 1.0 (ref) | 1.10 (1.09–1.12) | 1.27 (1.25–1.29) | 1.39 (1.35–1.42) | 1.51 (1.46–1.57) | 1.89 (1.87–1.92) | 1.54 (1.52–1.56) |
| **TIA/stroke** | 1.0 (ref) | 0.96 (0.95–0.97) | 1.02 (1.01–1.04) | 1.10 (1.08–1.13) | 1.20 (1.15–1.25) | 1.22 (1.20–1.23) | 1.25 (1.23–1.26) |
| **All-cause mortality** | 1.0 (ref) | 0.80 (0.79–0.81) | 0.88 (0.88–0.89) | 1.07 (1.06–1.09) | 1.43 (1.39–1.46) | 1.44 (1.43–1.45) | 1.57 (1.56–1.58) |

Data shown are hazard ratios with 95% confidence intervals for each BMI group relative to the reference group (normal weight), for men relative to women and for ever smokers relative to never smokers, derived from Cox proportional hazard models adjusted for age, sex and smoking status.

BMI, body mass index; HR, hazard ratio; MI, myocardial infarction; TIA, transient ischemic attack.

**Table S4** Risks for each condition and event in supplementary analyses adjusted for baseline comorbidities.

| Condition/ event | BMI group (kg/m$^2$) | | | | | Sex (HRs for men, relative to women) | Smoking (HRs for ever, relative to never) | Baseline comorbidity | | | |
|---|---|---|---|---|---|---|---|---|---|---|---|
| | Normal | 25–29.9 | 30–34.9 | 35–39.9 | 40–45 | | | Type 2 diabetes | Hypertension | Dyslipidaemia | CV event |
| **Type 2 diabetes** | 1.0 (ref) | 2.30 (2.27–2.34) | 4.73 (4.65–4.80) | 7.81 (7.67–7.96) | 10.8 (10.5–11.0) | 1.49 (1.48–1.51) | 1.22 (1.20–1.23) | | 1.68 (1.67–1.70) | 1.28 (1.26–1.30) | 1.21 (1.19–1.23) |
| **Asthma** | 1.0 (ref) | 1.27 (1.25–1.30) | 1.50 (1.47–1.53) | 1.68 (1.63–1.73) | 1.78 (1.70–1.86) | 0.71 (0.70–0.72) | 1.26 (1.24–1.28) | 0.91 (0.88–0.94) | 1.22 (1.20–1.25) | 1.02 (1.00–1.05) | 1.17 (1.12–1.21) |
| **Sleep apnea** | 1.0 (ref) | 2.17 (2.08–2.27) | 4.88 (4.68–5.09) | 9.88 (9.45–10.3) | 18.3 (17.4–19.2) | 3.13 (3.05–3.21) | 1.27 (1.24–1.30) | 1.03 (0.98–1.07) | 1.25 (1.21–1.28) | 1.26 (1.22–1.30) | 1.00 (0.95–1.05) |
| **Osteoarthritis** | 1.0 (ref) | 1.33 (1.32–1.34) | 1.64 (1.62–1.66) | 1.93 (1.90–1.96) | 2.23 (2.18–2.28) | 0.68 (0.67–0.68) | 1.03 (1.02–1.04) | 0.94 (0.92–0.95) | 1.16 (1.15–1.17) | 1.06 (1.05–1.07) | 1.04 (1.02–1.05) |
| **Heart failure** | 1.0 (ref) | 1.06 (1.05–1.07) | 1.40 (1.38–1.42) | 1.99 (1.95–2.04) | 2.80 (2.71–2.89) | 1.39 (1.38–1.41) | 1.35 (1.33–1.36) | 1.56 (1.54–1.59) | 1.57 (1.55–1.59) | 1.04 (1.03–1.05) | 1.91 (1.88–1.94) |
| **Chronic kidney disease** | 1.0 (ref) | 1.17 (1.16–1.18) | 1.34 (1.33–1.36) | 1.49 (1.47–1.52) | 1.69 (1.65–1.73) | 0.84 (0.83–0.85) | 1.10 (1.09–1.11) | 1.69 (1.68–1.71) | 1.93 (1.92–1.95) | 1.14 (1.13–1.15) | 1.37 (1.36–1.39) |
| **Hypertension** | 1.0 (ref) | 1.41 (1.40–1.42) | 1.91 (1.89–1.93) | 2.46 (2.43–2.49) | 2.95 (2.90–3.01) | 1.65 (1.64–1.66) | 1.11 (1.10–1.12) | 2.34 (2.32–2.37) | | 1.24 (1.23–1.25) | 1.56 (1.54–1.58) |
| **Dyslipidaemia** | 1.0 (ref) | 1.34 (1.34–1.35) | 1.58 (1.57–1.60) | 1.73 (1.71–1.75) | 1.82 (1.80–1.85) | 1.35 (1.34–1.36) | 1.27 (1.27–1.28) | 3.12 (3.10–3.15) | 1.87 (1.86–1.88) | | 2.31 (2.28–2.33) |
| **Atrial fibrillation** | 1.0 (ref) | 1.11 (1.10–1.13) | 1.41 (1.38–1.43) | 1.83 (1.79–1.88) | 2.47 (2.39–2.56) | 1.50 (1.48–1.52) | 1.06 (1.05–1.07) | 0.96 (0.94–0.98) | 1.56 (1.54–1.58) | 0.97 (0.96–0.99) | 1.28 (1.26–1.30) |
| **Unstable angina/MI** | 1.0 (ref) | 1.03 (1.02–1.04) | 1.11 (1.09–1.12) | 1.14 (1.12–1.17) | 1.18 (1.14–1.23) | 1.66 (1.64–1.68) | 1.42 (1.41–1.44) | 1.53 (1.50–1.55) | 1.54 (1.52–1.56) | 1.26 (1.25–1.28) | 2.47 (2.43–2.51) |
| **TIA/stroke** | 1.0 (ref) | 0.92 (0.91–0.94) | 0.94 (0.92–0.95) | 0.98 (0.95–1.00) | 1.02 (0.98–1.06) | 1.11 (1.10–1.13) | 1.20 (1.19–1.21) | 1.42 (1.39–1.44) | 1.38 (1.37–1.40) | 0.92 (0.91–0.93) | 2.17 (2.14–2.20) |

| | | | | | | | | | | | |
|---|---|---|---|---|---|---|---|---|---|---|---|
| **All-cause mortality** | 1.0 (ref) | 0.77 (0.76–0.77) | 0.81 (0.80–0.82) | 0.95 (0.94–0.97) | 1.21 (1.18–1.24) | 1.36 (1.35–1.37) | 1.54 (1.53–1.55) | 1.54 (1.53–1.56) | 1.36 (1.35–1.37) | 0.88 (0.87–0.88) | 1.60 (1.59–1.62) |

Data shown are hazard ratios with 95% confidence intervals for each BMI group relative to the reference group (normal weight), for men relative to women, for ever smokers relative to never smokers, and for individuals with type 2 diabetes, hypertension, dyslipidaemia or previous CV event relative to individuals without those baseline comorbidities. Data are derived from Cox proportional hazard models adjusted for age, sex, smoking status and presence of high-prevalence baseline comorbidities. Baseline CV events considered were unstable angina, MI, TIA and stroke.

BMI, body mass index; CV, cardiovascular; MI, myocardial infarction; TIA, transient ischemic attack.
